# Synthesis of a 3,7-Disubstituted Isothiazolo[4,3-*b*]pyridine as a Potential Inhibitor of Cyclin G-Associated Kinase

**DOI:** 10.3390/molecules29050954

**Published:** 2024-02-22

**Authors:** Tom Grisez, Nitha Panikkassery Ravi, Mathy Froeyen, Dominique Schols, Luc Van Meervelt, Steven De Jonghe, Wim Dehaen

**Affiliations:** 1 Department of Chemistry, Sustainable Chemistry for Metals and Molecules, KU Leuven, Celestijnenlaan 200F, B-3001 Leuven, Belgium; tom.grisez@kuleuven.be (T.G.); nitha.panikkasseryravi@kuleuven.be (N.P.R.); 2 Laboratory of Medicinal Chemistry, Rega Institute for Medical Research, Department of Pharmaceutical and Pharmacological Sciences, KU Leuven, Herestraat 49, P.O. Box 1041, B-3000 Leuven, Belgium; mathy.froeyen@kuleuven.be; 3 Laboratory of Virology and Chemotherapy, Rega Institute for Medical Research, Department of Microbiology, Immunology and Transplantation, KU Leuven, Herestraat 49, P.O. Box 1043, B-3000 Leuven, Belgium; dominique.schols@kuleuven.be (D.S.); steven.dejonghe@kuleuven.be (S.D.J.); 4 Department of Chemistry, Biomolecular Architecture, KU Leuven, Celestijnenlaan 200F, B-3001 Leuven, Belgium; luc.vanmeervelt@kuleuven.be

**Keywords:** GAK, isothiazolo[4,3-*b*]pyridine, kinase, inhibitor

## Abstract

Disubstituted isothiazolo[4,3-*b*]pyridines are known inhibitors of cyclin G-associated kinase. Since 3-substituted-7-aryl-isothiazolo[4,3-*b*]pyridines remain elusive, a strategy was established to prepare this chemotype, starting from 2,4-dichloro-3-nitropyridine. Selective C-4 arylation using ligand-free Suzuki-Miyaura coupling and palladium-catalyzed aminocarbonylation functioned as key steps in the synthesis. The 3-*N*-morpholinyl-7-(3,4-dimethoxyphenyl)-isothiazolo[4,3-*b*]pyridine was completely devoid of GAK affinity, in contrast to its 3,5- and 3,6-disubstituted congeners. Molecular modeling was applied to rationalize its inactivity as a GAK ligand.

## 1. Introduction

The Numb-associated kinase (NAK) family of serine/threonine kinases consists of four members in mammals: the adaptor-associated kinase I (AAK1), myristoylated and palmitoylated serine/threonine kinase I (MPSK1/STK16), BMP-2-induced kinase (BMP2K/BIKE), and cyclin G-associated kinase (GAK) [1]. The different NAKs are involved in a broad array of cellular activities, including *trans*-Golgi network (TGN) protein secretion [2,3,4], cell proliferation and differentiation [5], as well as cell division through mitosis [6].

GAK, specifically, is essential for clathrin-mediated endocytosis and plays an important role in the *trans*-Golgi network to lysosome trafficking [7,8]. GAK shares some functions with AAK1, including the ability to phosphorylate the adaptor protein 2 (AP2) µ–subunit [2]. This enhances the binding affinity of this unit for tyrosine- and dileucine-based sorting signals on receptor proteins in the plasma membrane [9,10]. Phosphorylation of the APs by GAK enhances their coupling to cargo proteins and recruits clathrin to the membrane to first form clathrin-coated pits and then clathrin-coated vesicles (CCVs) [7,11,12,13,14,15,16]. GAK is also involved in the uncoating of CCVs in the cytoplasm [7,17].

**Figure 1:**
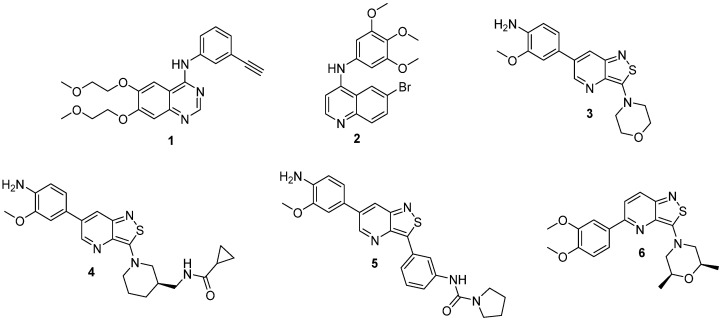
Previously reported GAK inhibitors.

It has been demonstrated that GAK is required in the early and late stages of the lifecycle of various RNA viruses, such as the hepatitis C virus [18], the dengue virus, and the Ebola virus [19]. Hence, GAK has been designated as a “master regulator” of viral infection and is being pursued as a host target for the development of broad-spectrum antiviral agents. A repurposing approach has led to the identification of erlotinib (compound **1**, Figure 1), an FDA-approved drug for the treatment of non-small-cell lung cancer, as a potent GAK inhibitor [8]. Erlotinib is endowed with broad-spectrum in vitro antiviral activity and displays in vivo activity in a preclinical murine model of hepatitis C virus infection [20]. The main pharmacological target of erlotinib is the epidermal growth factor receptor (EGFR), and its high GAK affinity is actually an off-target effect. Therefore, erlotinib is not an ideal probe to study GAK-related biology. In order to come up with potent, selective, and drug-like GAK inhibitors, two different chemotypes have been developed.

Several 4-anilinoquinolines and 4-anilinoquinazolines have been described with a nanomolar binding affinity to GAK and varying levels of kinase selectivity [21,22,23,24,25]. From this research, SGC-GAK-1 (compound **2**, Figure 1) emerged as an excellent chemical probe for GAK. SGC-GAK-1 displays a potent GAK affinity, with an IC_50_ value of 48 nM in a GAK live cell target engagement assay and was over 50-fold selective for GAK when evaluated in a panel of more than 400 human kinases. 

Our group has instead focused on isothiazolo[4,3-*b*]pyridine chemistry for the development of GAK inhibitors as broad-spectrum antiviral agents. The first series of isothia-zolo[4,3-*b*]pyridines carried a substituted aryl (usually a 3,4-dimethoxyphenyl or a 3-methoxy-4-amino-phenyl) at position 6 of the central scaffold, with structural modifications at position 3 [26]. GAK tolerated a considerable amount of structural variety in this position, ranging from saturated cycloaliphatic heterocycles (e.g., a morpholinyl, as found in compound **3**, and a piperidinyl, as found in compound **4**) to substituted phenyl rings (e.g., compound **5**) [27]. More recently, the aryl group was moved from position 6 to position 5, yielding a series of 3,5-disubstituted isothiazolo[4,3-*b*]pyridines, exemplified by compound **6** [28]. Isothiazolo[4,3-*b*]pyridines **3**–**6** display potent GAK affinity and are endowed with cellular activity against various viruses [26,27,28].

In this work, the SAR of isothiazolo[4,3-*b*]pyridines as GAK inhibitors was expanded by moving the aryl group to position 7 of the scaffold, yielding a hitherto-unknown 3,7-disubstituted isothiazolo[4,3-*b*]pyridine. A 3,4-dimethoxyphenyl substituent was selected as the substituent at position 7, whereas, at position 3, a morpholinyl moiety was introduced, as this substitution pattern allows for a side-by-side comparison with the corresponding known 3,5- and 3,6-disubstituted isothiazolo[4,3-*b*]pyridines.

## 2. Results and Discussion

### 2.1. Chemistry

**Figure 2:**
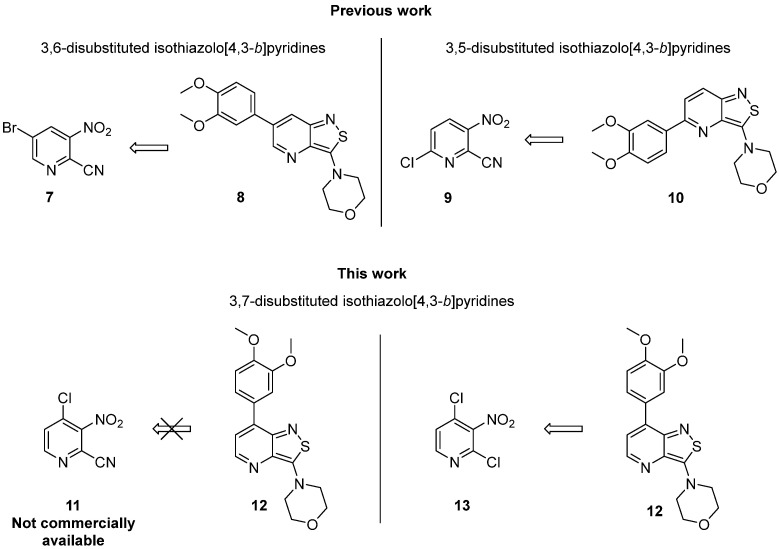
Retrosynthesis of various isothiazolo[4,3-*b*]pyridines.

Previously, 3,6- (compound **8**) and 3,5-disubstituted (compound **10**) isothiazolo[4,3-*b*]pyridines were prepared from the commercially available halogenated 3-nitropicolinonitrile building blocks **7** and **9**, respectively (Figure 2) [26,28]. In analogy, to access a 3,7-disubstituted isothiazolo[4,3-*b*]pyridine (compound **12**), the availability of a C-4 halogenated 3-nitropicolinonitrile **11** is required. However, 4-halo-pyridines are not readily commercially available.

**Scheme 1:**
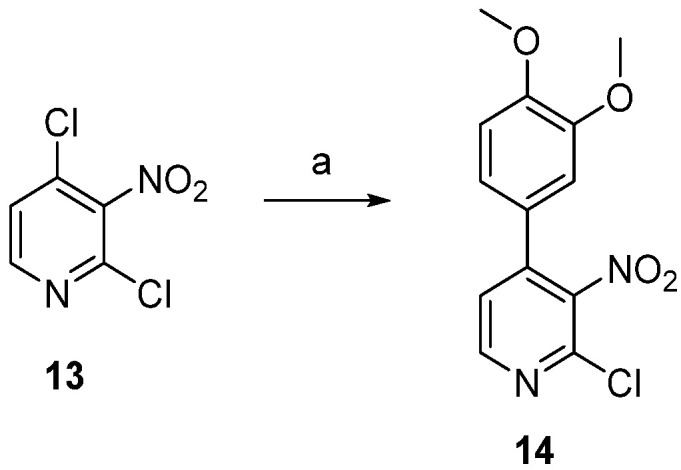
Synthesis of 2-chloro-3-nitro-4-(3,4-dimethoxyphenyl)pyridine (**14**). Reagents and conditions: (a) PdCl_2_, NBu_4_Br, Na_2_CO_3_, DMF/water 20:1, 110 °C, 17 h.

An alternative strategy was pursued starting from 2,4-dichloro-3-nitropyridine **13**, which is a commercially available and inexpensive building block. The expected higher reactivity at the 2 position was circumvented using recently reported ligand-free Suzuki-Miyaura coupling under “Jeffery-Heck” reaction conditions [29]. Applying these reaction circumstances (using PdCl_2_ as the palladium salt, Na_2_CO_3_ as the inorganic base, and NBu_4_Br as the tetraalkylammonium additive) yielded the 4-arylated compound **14** in 66% yield (Scheme 1).

**Figure 3:**
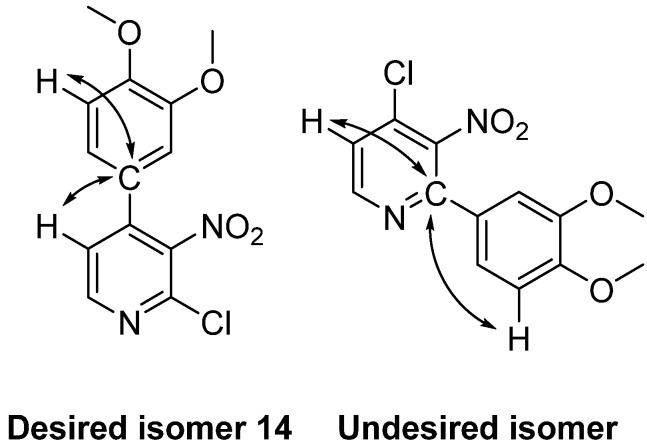
Long-range C-H couplings in the two possible isomers.

The product was initially characterized by ^1^H- and ^13^C-NMR spectroscopy, which was not conclusive due to the similarity in the expected signals of the two isomers. HMBC experiments seemed to support the formation of the desired isomer **14**, as two long-range couplings were observed between a central carbon atom and two hydrogen atoms on the two aryl rings, as shown in Figure 3. This configuration would be more likely in the desired isomer.

**Figure 4:**
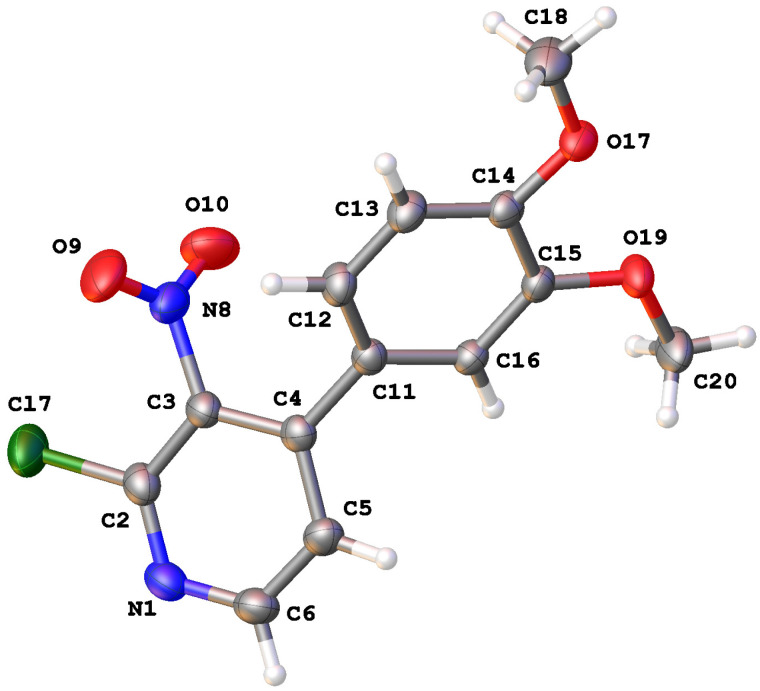
View of the crystal structure of compound **14**, showing the numbering scheme and thermal ellipsoids at the 30% probability level.

As the designation of the regio-chemistry relied on the possibly ambiguous assignment of proton and carbon signals, the structure of **14** was confirmed by single-crystal X-ray crystallography (CCDC 2308765, Figure 4). The dihedral angle between both rings was 49.82(9)°. The best plane through the nitro group made an angle with the pyridine ring of 74.61(3)°.

**Scheme 2:**
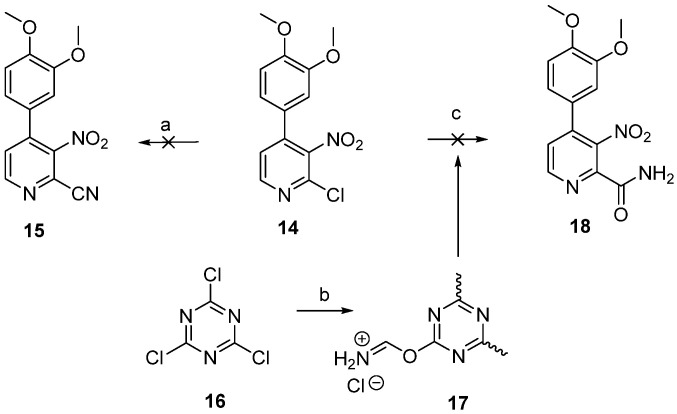
Synthesis of a 3-nitro-4-aryl-picolinamide **18**. Reagents and conditions: (a) Cu(I)CN, DMF, 90 °C, 24 h; (b) formamide, rt, 2 h; and (c) Pd(OAc)_2_, dppf, DMF, 140 °C, 24 h.

The remaining chlorine at position 2 of the pyridine scaffold was exploited to introduce a nitrile group via a Rosenmund–Von Braun reaction (Scheme 2). This type of cyanation reactions typically requires harsh reaction conditions (150–280 °C) and usually starts from aryl iodides or aryl bromides. However, it has been shown that 2-chloro-6-methoxy-3-nitropyridine can be converted into 6-methoxy-3-nitropyridine-2-carbonitrile under rather mild reactions conditions (three equivalents of copper(I)cyanide in DMF at 90 °C for 5 h) [30]. Unfortunately, applying these reaction conditions to substrate **14** did not lead to the formation of the desired product **15**, even after extensive heating for 24 h. Alternative cyanation procedures in which the cyanide anion was produced in situ from 2,4,6-trichloro-1,3,5-triazine (cyanuric chloride **16**) and formamide in the presence of a base and then coupled via a heterogeneous palladium catalyst also did not afford the desired compound **15** [31].

The difficulties associated with the introduction of the nitrile moiety and the fact that, ultimately, a carbothioamide group needs to be installed at position 2 of the scaffold led us to redirect our efforts towards an aminocarbonylation reaction. The Pd-catalyzed aminocarbonylation of an aryl halide using a mixture of cyanuric chloride and dimethylformamide yielding a reactive amidating species has been shown previously to allow the installation of dimethylformamide on a (hetero)aryl scaffold [32]. Employing similar reaction conditions (i.e., using cyanuric chloride, formamide, and Pd(OAc)_2_/dppf as the catalytic system in DMF at 140 °C) on the 2-chloro-pyridine analogue **14** in an attempt to form the primary amide was unsuccessful, and no desired product **18** was isolated.

Therefore, alternative strategies to install the primary carboxamide moiety onto 2-chloropyridine **14** were explored. Carbon monoxide was generated in a two-chamber reactor by means of the reaction of mesyl chloride, formic acid, and triethylamine following a reported procedure [33]. Unfortunately, the different palladium-catalyzed reactions that were evaluated for the carbonylation of 2-chloropyridine **14** were unsuccessful. These included a phosphine-free approach using methoxyamine as the ammonia source and iodide as the palladium ligand [34] and a method using triphenylphosphine as the ligand, DMAP as the base, and formamide as the amine source [35].

Since the introduction of a primary amide was cumbersome, benzylamine was used as the amine source, as the benzyl group could theoretically be removed simultaneously with the reduction of the nitro group in the subsequent step. For the introduction of the benzylamide substituent by means of a palladium-catalyzed aminocarbonylation, Pd(OAc)_2_/Xantphos was selected as the catalytic system, sodium carbonate as the base, benzylamine as the amine, and toluene as the solvent, with CO gas generated in situ from mesyl chloride, formic acid, and triethylamine in a two-chamber reactor following a reported procedure [33]. Under these conditions (Entry 1, Table 1), the yield of the desired compound **20** was 23%, and the 2-benzylaminopyridine analogue **21** was also formed in 15% yield. Increasing the number of equivalents of the CO-surrogates from 1.3 to 2.6 (entries 1 and 2, respectively) improved the yield of compound **20** from 23% to 42%. To our delight, increasing the catalytic loading from 1% to 2.5% allowed us to further increase the yield of the desired compound **20** to 78%.

The catalytic hydrogenation of **20** using Pd/C and hydrogen gas yielded the complete reduction of the nitro to the corresponding amino group but did not cleave off the benzyl group. Alternative methods to remove the benzyl moiety, such as the treatment of **20** with Lewis and Brønsted–Lowry acids or oxidative cleavage with *N*-bromosuccinimide (NBS) and *N*-methylacetamide (NMA), were equally unsuccessful [36].

**Scheme 3:**
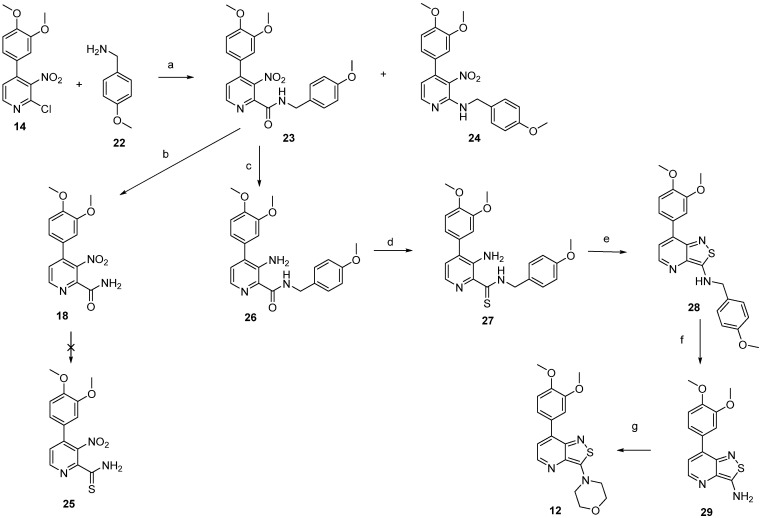
Synthesis of isothiazolo[4,3-*b*]pyridine **12**. Reagents and conditions: (a) chamber A: Na_2_CO_3_, Pd(OAc)_2_, XantPhos, toluene; chamber B: HCOOH, MsCl, Et_3_N, toluene, 100 °C, 2.5 h; (b) CAN, ACN/water, 80 °C, 3 h or TFA, 70 °C, 16 h; (c) Fe powder, AcOH, 70 °C, 20 h; (d) Lawesson’s reagent, toluene, 100 °C, 2 h; (e) H_2_O_2_ (30% in water), DCM/MeOH, 40 °C, 18 h; (f) DDQ, DCM/water, RT, 4 h; and (g) bis(2-bromodiethyl)ether, K_2_CO_3_, DMF, 100 °C, 3 h.

It was, therefore, decided to switch to the more electron-rich *para*-methoxybenzyl (PMB) protecting group, since this group had been anticipated to be easier to remove. Hence, the aminocarbonylation of **14** with *p*-methoxybenzylamine as the amine source using the previously optimized reaction conditions (i.e., 2.6 eq. of CO gas-surrogates and a catalyst loading of 2.5 mol%) yielded the desired compound **23** in 67% yield, along with a minor amount (6%) of side product **24** (Scheme 3). Oxidative cleavage with cerium ammonium nitrate (CAN) or acidic deprotection with trifluoroacetic acid (TFA) of the PMB-protecting group of intermediate **23** gave the 3-nitropyridine derivative **18** in good yield (84% and 75%, respectively). Unfortunately, the subsequent thionation of the amide moiety with phosphorus pentasulfide or Lawesson’s reagent did not provide compound **25**. Therefore, the nitro group of intermediate **23** was first reduced by treatment with iron in acetic acid giving the 3-amino-pyridine analogue **26** in 89% yield. Then, treatment with Lawesson’s reagent in toluene afforded thioamide **27** in 51% yield. The work-up of this thionation reaction included refluxing the crude reaction mixture in a concentrated sodium bicarbonate solution to destroy the side products of the Lawesson’s reagent, which otherwise hampered the purification. Oxidative cyclization of the protected thioamide **27** provided the 3,7-disubstituted isothiazolo[4,3-*b*]pyridine **28** in 85% yield. The previously successful methods of deprotection of the PMB-group (CAN and TFA) afforded very complex reaction mixtures and low yields of the product on this substrate. Alternatively, oxidative deprotection using 2,3-dichloro-5,6-dicyano-1,4-benzoquinone (DDQ) afforded the 3-amino-isothiazolo[4,3-*b*]pyridine **29** in moderate yield (44%) in a much cleaner reaction. Finally, the exocyclic amino group was cyclized into a morpholine by means of treatment with bis(2-bromoethyl) ether in a diluted reaction mixture, affording target compound **12** in 52% yield.

### 2.2. GAK Affinity Studies

The 3,7-disubstituted isothiazolo[4,3-*b*]pyridine analogue **12** was evaluated for its GAK affinity in a LanthaScreen^®^ Eu GAK binding assay [37], which is based on the displacement of a proprietary Alexa Fluor^®^ 647-labeled, ATP-competitive GAK inhibitor known as a kinase tracer. When both the tracer and a europium-labeled anti-tag antibody are bound to GAK, a high degree of FRET (fluorescence resonance energy transfer) is observed from the europium (Eu) donor fluorophore to the Alexa Fluor^®^ 647 acceptor fluorophore on the kinase tracer. An effective GAK inhibitor should displace the tracer, which would decrease the amount of observed FRET, which allows for the determination of the IC_50_ value.

The 3,7-disubstituted isothiazolo[4,3-*b*]pyridine analogue **12** completely lacked GAK affinity. This is in sharp contrast with the two other regio-isomers (i.e., the 3,5 and 3,6-disubstituted isothiazolo[4,3-*b*]pyridines **10** and **8**) that did show potent GAK binding in the LanthaScreen assay (Table 2).

### 2.3. Molecular Modeling

**Figure 5:**
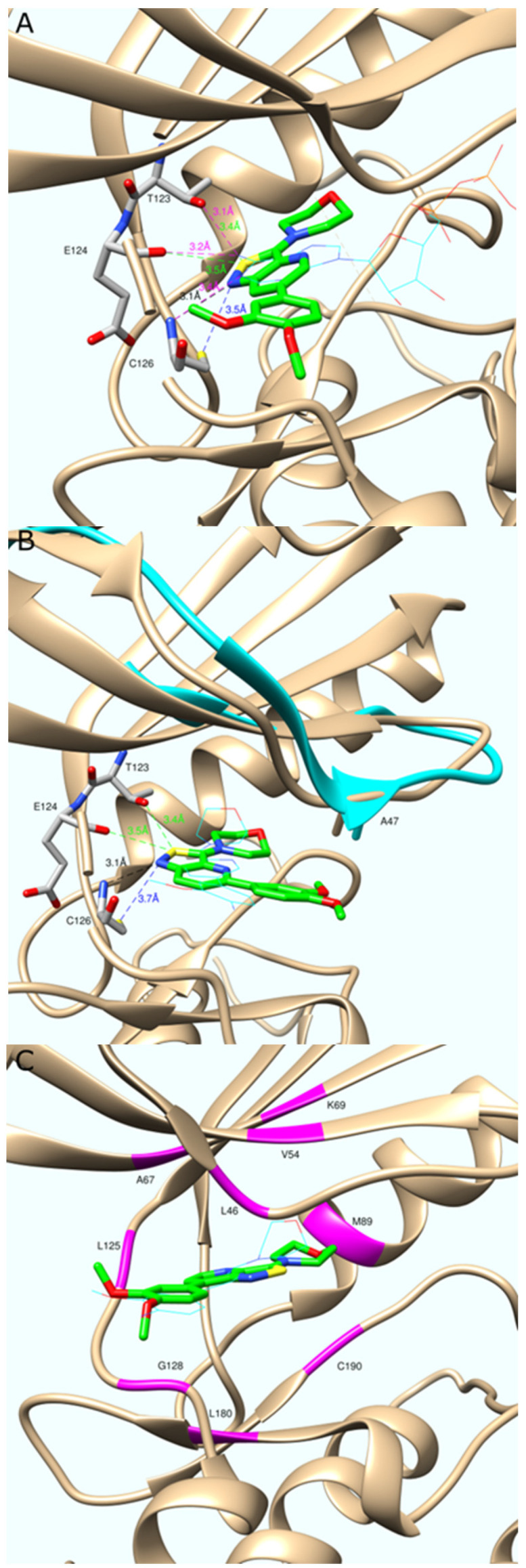
(**A**) Isothiazolo[4,3-*b*]pyridine **8** binds in a similar way to isothiazolo[4,3-*b*]pyridine **3** and is shown together with superimposed ATP (wire) from PDB entry 1ATP. Hydrogen bonds from ATP to the hinge region of GAK are shown in magenta. The hydrogen bond from compound **8** is shown in black, the ChB interactions in green, and the SH-N-hydrogen bond in blue. (**B**) Isothiazolo[4,3-*b*]pyridine **10** docks in the same plane as isothiazolo[4,3-*b*]pyridine **3**, maintaining the same interactions as compound **8**, if an induced fit is allowed for the loop containing residue A47 to prevent a clash with the A47 sidechain. The original loop orientation is shown in the cyan ribbon. Reference isothiazolo[4,3-*b*]pyridine **3** from PDB entry 4Y8D is shown in wire. (**C**) Isothiazolo[4,3-*b*]pyridine **12** docks in the same plane as isothiazolo[4,3-*b*]pyridine **3** but rotated and shifted, with no specific interactions. Residues having possible Van der Waals contact with this inhibitor are labeled and colored in magenta. The compound from PDB entry 4Y8D is shown in wire for reference.

To rationalize the lack of GAK affinity of the novel derivative **12**, the three regio-isomeric isothiazolo[4,3-*b*]pyridines were submitted for a docking analysis using the experimentally determined co-crystal structure of compound **3** with GAK (PDB 4Y8D). As expected, the 3,6-disubstituted derivative **8** adopted a binding mode that is very similar to that of compound **3** in 4Y8D (Figure 5a). This means that compound **8** acts as a typeI kinase inhibitor, binding to the active conformation (also known as DFG-in) of GAK in the ATP-binding pocket. Key interactions between the isothiazolo[4,3-*b*]pyridine core of compound **8** and the hinge region of GAK include a hydrogen bond between the nitrogen of the isothiazole moiety and both the backbone NH and sidechain SH of Cys126 [38], along with a chalcogen bond (ChB) interaction [39] between the sulfur of the isothiazole ring and both the backbone oxygen of Glu124 and the alcoholic oxygen of Thr123. These residues’ backbones would normally form hydrogen bonds with the amine and N1 of adenosine (Figure 5a). The 3,4-dimethoxyphenyl moiety of compound **8** is oriented towards the solvent. The morpholinyl moiety contributes to binding by means of Van der Waals interactions with the sidechain atoms of residues Val54, Ala67, and Thr123.

The initial prediction of the binding mode by docking revealed that the 3,5-disubstituted analogue **10** did not dock in a similar way to compound **8**, prevented by a steric clash between the *para*-methoxy group and the sidechain of Ala47. Careful examination of the existing GAK structures in the PDB database indicated that the loop with amino acids 38–57 is flexible. Superimposition of this loop from PDB entry 4C57 [4] onto the 4Y8D structure and repeating the docking resulted in a similar orientation of compound **10** to compound **8** in the binding pocket, maintaining all important interactions with the hinge region of GAK. (Figure 5b). This explains the similar activities of compounds **8** and **10** as GAK binders. The software also docked the 3,7-disubstituted analogue **12** in the same plane as the parent analogue **3** but with the isothiazole moiety rotated and shifted away from the hinge region so that no observable directional interactions were formed between the central scaffold of compound **12** and the kinase, which explained the lack of GAK affinity of isothiazolo[4,3-*b*]pyridine **12**.

## 3. Materials and Methods

### 3.1. Chemistry

The chemicals were obtained from Acros Organics (Geel, Belgium), Alfa Aesar (Lancashire, UK), BLD Pharma (Reinbek, Germany), Fluorochem (Glossop, UK), J&K Scientific (Lommel, Belgium), Merck/Sigma Aldrich (Darmstadt, Germany), and TCI Chemicals (Zwijndrecht, Belgium) and were used without further purification unless otherwise specified. NMR measurements were performed on a Bruker Avance III HD 400 MHz or a Bruker Avance II+ 600 MHz, and chemical shifts (δ) were reported in parts per million (ppm) in reference to tetramethylsilane or the internal NMR solvent signal. Coupling constants (J) were reported in hertz (Hz). HR-MS spectra were acquired on a quadrupole orthogonal acceleration time-of-flight mass spectrometer (Synapt G2 HDMS, Waters, Milford, MA, USA). The samples were infused at 3 µL/min, and the spectra were obtained in a positive ionization mode with a resolution of 15000 (FWHM), using leucine enkephalin as the lock mass. Melting points were measured on a Reichert-Jung Thermovar system and are uncorrected. TLC analysis was performed on TLC aluminum sheets bought from Sigma Aldrich (pore diameter of 60 Å, fluorescent indicator at 254 nm). The products were visualized by UV irradiation at 254 or 365 nm or under visible light. Homemade two-chamber reactors were modeled after the COware design by the Skrydstrup group [40].

#### 3.1.1. 2-Chloro-4-(3,4-dimethoxyphenyl)-3-nitropyridine (**14**)

To a 250 mL two-necked round-bottom flask (RBF) equipped with a stir bar were added PdCl_2_ (0.138 g, 0.777 mmol, 0.05 equiv.), 2,4-dichloro-3-nitropyridine (3.000 g, 15.5 mmol, 1.0 equiv.), 3,4-dimethoxyphenylboronic acid (3.112 g, 17.1 mmol, 1.1 equiv.), Na_2_CO_3_ (4.943 g, 46.6 mmol, 3.0 equiv.), and NBu_4_Br (25.058 g, 77.7 mmol, 5.0 equiv.); a condenser was attached; and the apparatus was then purged with nitrogen gas for 15 min in a sonication bath. To this were added DMF (62.2 mL) and water (1.5 mL), both purged separately with nitrogen gas for 15 min. The RBF was heated in an oil bath at 110 °C for 17 h under a nitrogen atmosphere. The apparatus was then left to cool. A total of 400 mL of water was added, and the products were extracted five times into EtOAc (125 mL). The organic layers were combined and washed five times with water and once with conc. brine (75 mL). The organic layers were dried with anhydrous sodium sulfate and filtered, and solvent was removed in vacuo. The product could be recrystallized from EtOH as orange crystals (2.95 g, 10.0 mmol, 64%) or, for smaller quantities, purified via silica gel column chromatography using petroleum ether (PE)/EtOAc (7:3). The title compound was, in this case, isolated as a yellow powder (1.12 g, 3.4 mmol, 66%).

**MP** 155.8–158.3 °C. **^1^H NMR** (400 MHz, CDCl_3_) δ 8.49 (d, *J* = 5.1 Hz, 1H), 7.37 (d, *J* = 5.1 Hz, 1H), 6.97 (dd, *J* = 8.3, 2.0 Hz, 1H), 6.93 (d, *J* = 8.3 Hz, 1H), 6.88 (d, *J* = 2.0 Hz, 1H), 3.91 (s, 3H), 3.88 (s, 3H).**^13^C NMR** (101 MHz, CDCl_3_) δ 151.0, 150.0, 149.6, 145.2, 144.4, 142.3, 125.2, 124.3, 120.8, 111.8, 110.5, 56.13, 56.12. **HR-MS** (ESI-Q-TOF): m/z[M+H]^+^ C_13_H_11_ClN_2_O_4_ Calculated: 295.0480. Measured: 295.0484.

#### 3.1.2. 4-(3,4-Dimethoxyphenyl)-3-nitropicolinamide (**18**)

Two procedures were used.

**Procedure A**: To a vial with a screw cap equipped with a stir bar was added **23** (50.0 mg, 118.1 µmol, 1.0 equiv.), followed by CAN (323.7 mg, 0.59 mmol, 5.0 equiv.), water (1.5 mL), and ACN (1.5 mL). The vial was heated at reflux for 3 h. Water (10 mL) was added, and the product was extracted three times into EtOAc (15 mL). The organic layers were combined, washed once with brine, dried with anhydrous sodium sulfate, and filtered. The solvent was removed in vacuo. The product was purified via silica gel column chromatography using PE/EtOAc (2:8). The title compound was isolated as slightly yellow flakes (30.0 mg, 0.1 mmol, 84%).

**Procedure B**: To an RBF (10 mL) equipped with a stir bar was added **23** (20.0 mg, 0.047 mmol, 1.0 equiv.), followed by TFA (0.47 mL). A condenser was attached, and the RBF was placed in an oil bath at 70 °C for 16 h. The RBF was allowed to cool, and a conc. sodium bicarbonate solution was added until neutralized. The products were extracted three times into EtOAc (10 mL). The organic layers were combined and washed with brine, dried with anhydrous sodium sulfate, and filtered. The solvent was removed in vacuo. The product was purified via silica gel column chromatography using PE/EtOAc (4:6). The title compound was isolated as slightly yellow flakes (10.3 mg, 0.033 mmol, 71%). The solubility of this compound in various NMR solvents was extremely low, and, as such, only a ^1^H NMR spectrum could be collected.

**MP** 254.4–257.4 °C. **^1^H NMR** (400 MHz, DMSO-*d*_6_) δ 8.82 (d, *J* = 5.0 Hz, 1H), 8.39 (s, 1H), 8.00 (s, 1H), 7.85 (d, *J* = 5.0 Hz, 1H), 7.09 (d, *J* = 8.4 Hz, 1H), 7.02 (d, *J* = 2.2 Hz, 1H), 6.94 (dd, *J* = 8.3, 2.2 Hz, 1H), 3.81 (s, 3H), 3.78 (s, 3H).

#### 3.1.3. *N*-Benzyl-4-(3,4-dimethoxyphenyl)-3-nitropicolinamide (**20**)

To chamber A of a flame-dried two-chamber reactor (10 mL) equipped with a stir bar in each chamber were added Pd(OAc)_2_ (1.0 mg, 4.5 µmol, 0.026 equiv.), XantPhos (3.0 mg, 5.2 µmol, 0.031 equiv.), Na_2_CO_3_ (53.9 mg, 0.51 mmol, 3.0 equiv.), and **14** (50.0 mg, 0.17 mmol, 1.0 equiv.). The apparatus was purged with nitrogen gas for 15 min. All the following reagents were added via a syringe. To chamber B was added dry nitrogen-purged toluene (2 mL). To this were added mesyl chloride (0.034 mL, 0.44 mmol, 2.6 equiv.) and formic acid (0.019 mL, 0.44 mmol, 2.6 equiv.). To chamber A were added dry nitrogen-purged toluene (0.5 mL) and *para*-methoxybenzylamine (27.3 mg, 0.25 mmol, 1.5 equiv.). Et_3_N (0.12 mL, 0.88 mmol, 5.2 equiv.) was added dropwise to chamber B at room temperature. After two minutes of stirring at room temperature, the two-chamber reactor was placed in an oil bath at 100 °C. After 2.5 h, the reactor was removed from the oil bath and allowed to cool fully. The reactor was vented while stirring for five minutes at room temperature. The contents were transferred to a separatory funnel; water (20 mL) was added; and the products were extracted three times into EtOAc (7.5 mL). The organic layers were combined and washed once with brine, dried with anhydrous sodium sulfate, and filtered. The solvent was removed in vacuo. The product was purified via silica gel column chromatography using PE/EtOAc (7:3–0:10). The title compound was isolated as a yellow powder (52.0 mg, 0.132 mmol, 78%).

**MP** 127.6–128.9 °C. **^1^H NMR** (400 MHz, CDCl_3_) δ 8.60 (d, *J* = 4.9 Hz, 1H), 8.25 (t, *J* = 6.0 Hz, 1H), 7.55 (d, *J* = 4.9 Hz, 1H), 7.36 (d, *J* = 4.4 Hz, 4H), 7.30 (td, *J* = 8.9, 8.5, 4.5 Hz, 1H), 6.97 (dd, *J* = 8.3, 2.1 Hz, 1H), 6.92 (d, *J* = 8.3 Hz, 1H), 6.90 (d, *J* = 2.1 Hz, 1H), 4.63 (d, *J* = 6.1 Hz, 2H), 3.91 (s, 3H), 3.88 (s, 3H).**^13^C NMR** (101 MHz, CDCl_3_) δ 160.9, 150.7, 149.4, 148.7, 145.8, 143.8, 140.6, 137.7, 128.9 (2C), 128.3, 128.1 (2C), 127.8, 125.4, 121.1, 111.6, 111.0, 56.12, 56.09, 43.7.

#### 3.1.4. *N*-Benzyl-4-(3,4-dimethoxyphenyl)-3-nitropyridin-2-amine (**21**)

The title compound was isolated as a side product of 20, eluting first off the column, isolated as a yellow oil (9.0 mg, 24.6 µmol, 15%).

**MP** 114.9–118 °C. **^1^H NMR** (400 MHz, CDCl_3_) δ 8.28 (d, *J* = 4.9 Hz, 1H), 7.42–7.27 (m, 5H), 6.99 (t, *J* = 5.7 Hz, 1H), 6.91 (d, *J* = 8.3 Hz, 1H), 6.88 (dd, *J* = 8.3, 1.9 Hz, 1H), 6.80 (d, *J* = 1.9 Hz, 1H), 6.62 (d, *J* = 4.9 Hz, 1H), 4.79 (d, *J* = 5.5 Hz, 2H), 3.92 (s, 3H), 3.88 (s, 3H). **^13^C NMR** (101 MHz, CDCl_3_) δ 151.8, 151.5, 149.9, 149.4, 147.1, 138.6, 130.3, 129.5, 128.9 (2C), 127.8 (2C), 127.6, 119.9, 115.1, 111.5, 110.4, 56.13, 56.07, 45.6.

#### 3.1.5. 4-(3,4-Dimethoxyphenyl)-*N*-(4-methoxybenzyl)-3-nitropicolinamide (**23**)

To chamber A of a flame-dried two-chamber reactor (100 mL) equipped with a stir bar in each chamber were added Pd(OAc)_2_ (10.0 mg, 0.045 mmol, 0.026 equiv.), XantPhos (30.0 mg, 0.052 mmol, 0.031 equiv.), Na_2_CO_3_ (539.5 mg, 5.1 mmol, 3.0 equiv.), and **14** (500.0 mg, 1.697 mmol, 1.0 equiv.). The apparatus was purged with nitrogen gas for 15 min. All the following reagents were added via a syringe. To chamber B was added dry nitrogen-purged toluene (20 mL). To this were added mesyl chloride (0.341 mL, 4.4 mmol, 2.6 equiv.) and formic acid (0.189 mL, 4.4 mmol, 2.6 equiv.). To chamber A were added dry nitrogen-purged toluene (5 mL) and *para*-methoxybenzylamine (349.1 mg, 2.5 mmol, 1.5 equiv.). Et_3_N (1.2 mL, 8.8 mmol, 5.2 equiv.) was added dropwise to chamber B at room temperature. After two minutes of stirring at room temperature, the two-chamber reactor was placed in an oil bath at 100 °C. After 2.5 h, the reactor was removed from the oil bath and allowed to cool fully. The reactor was vented while stirring for five minutes at room temperature. The contents were transferred to a separatory funnel; water (200 mL) was added; and the products were extracted three times into EtOAc (75 mL). The organic layers were combined and washed once with brine, dried with anhydrous sodium sulfate, and filtered. The solvent was removed in vacuo. The product was purified via silica gel column chromatography using PE/EtOAc (7:3–0:10). The title compound was isolated as a yellow powder (480.0 mg, 1.06 mmol, 67%).

**MP** 175.5–177.5 °C **^1^H NMR** (600 MHz, CDCl_3_) δ 8.59 (d, *J* = 4.9 Hz, 1H), 8.17 (t, *J* = 5.9 Hz, 1H), 7.54 (d, *J* = 4.9 Hz, 1H), 7.32–7.26 (m, 2H), 6.97 (dd, *J* = 8.3, 2.1 Hz, 1H), 6.93 (d, *J* = 8.3 Hz, 1H), 6.90 (d, *J* = 2.1 Hz, 1H), 6.90–6.86 (m, 2H), 4.57 (d, *J* = 5.9 Hz, 2H), 3.91 (s, 3H), 3.88 (s, 3H), 3.80 (s, 3H). **^13^C NMR** (151 MHz, CDCl_3_) δ 160.7, 159.2, 150.7, 149.3, 148.5, 145.7, 143.7, 140.6, 129.7, 129.5 (2C), 128.1, 125.3, 121.0, 114.2 (2C), 111.5, 110.9, 55.99, 55.96, 55.3, 43.1. **HR-MS** (ESI-Q-TOF): m/z[M+H]^+^ C_22_H_21_N_3_O_6_ Calculated: 424.1503. Measured: 424.1496.

#### 3.1.6. 4-(3,4-Dimethoxyphenyl)-*N*-(4-methoxybenzyl)-3-nitropyridin-2-amine (**24**)

The title compound was isolated as a side product of **23**, eluting first off the column, isolated as a yellow oil (31.0 mg, 78.4 µmol, 6%).

**MP** 89.5–91.2 °C. **^1^H NMR** (400 MHz, CDCl_3_) δ 8.28 (d, *J* = 4.9 Hz, 1H), 7.34–7.28 (m, 2H), 6.93–6.87 (m, 4H), 6.80 (d, *J* = 1.9 Hz, 1H), 6.61 (d, *J* = 4.9 Hz, 1H), 4.71 (d, *J* = 5.4 Hz, 2H), 3.91 (s, 3H), 3.88 (s, 3H), 3.81 (s, 3H). **^13^C NMR** (101 MHz, CDCl_3_) δ 159.2, 151.5, 151.2, 149.9, 149.3, 147.2, 130.5, 130.2, 129.34, 129.20 (2C), 119.9, 115.0, 114.2 (2C), 111.5, 110.4, 56.10, 56.04, 55.4, 45.2.

#### 3.1.7. 3-Amino-4-(3,4-dimethoxyphenyl)-*N*-(4-methoxybenzyl)picolinamide (**26**)

To a flame-dried vial with a screw cap equipped with a stir bar was added iron powder (263.8 mg, 4.7 mmol, 4.25 equiv.), followed by **23** (450.0 mg, 1.06 mmol, 1.0 equiv.) and AcOH (10.6 mL, 0.100 M). The vial was closed, brought under a nitrogen atmosphere, and then heated at 70 °C for 20 h. The solvent was removed in vacuo, and the remaining slurry was neutralized dropwise with a sodium bicarbonate solution until it reached a pH of 8. The product was extracted three times into EtOAc (75 mL). The organic layers were combined and washed three times with brine (20 mL) and then dried with anhydrous sodium sulfate and filtered. The solvent was removed in vacuo. The product was purified via silica gel column chromatography using PE/EtOAc (7:3). The title compound was isolated as an off-white powder (372.0 mg, 0.94 mmol, 89%) that fluoresced blue under UV light in solution.

**MP** 144.3–146.1 °C. **^1^H NMR** (400 MHz, CDCl_3_) δ 8.50 (t, *J* = 6.0 Hz, 1H), 7.85 (d, *J* = 4.5 Hz, 1H), 7.34–7.28 (m, 2H), 7.08 (d, *J* = 4.5 Hz, 1H), 6.99 (d, *J* = 2.1 Hz, 2H), 6.94 (d, *J* = 1.6 Hz, 1H), 6.91–6.85 (m, 2H), 6.27 (s, 2H), 4.56 (d, *J* = 6.0 Hz, 2H), 3.94 (s, 3H), 3.90 (s, 3H), 3.80 (s, 3H). **^13^C NMR** (101 MHz, CDCl_3_) δ 167.8, 158.9, 149.5, 149.3, 143.6, 136.7, 136.3, 130.7, 130.0, 129.1 (2C), 128.7, 127.4, 121.0, 114.1 (2C), 111.7, 111.5, 56.03, 56.01, 55.3, 42.6. **HR-MS** (ESI-Q-TOF): m/z[M+H]^+^ C_22_H_23_N_3_O_4_ Calculated: 394.1761. Measured: 394.1758.

#### 3.1.8. 3-Amino-4-(3,4-dimethoxyphenyl)-*N*-(4-methoxybenzyl)pyridine-2-carbothioamide (**27**)

To a flame-dried vial with a screw cap equipped with a stir bar was added **26** (372.0 mg, 0.94 mmol, 1.0 equiv.), followed by Lawesson’s reagent (1.800 g, 4.45 mmol, 4.7 equiv.) and dry nitrogen-purged toluene (14.8 mL), and was heated in an oil bath at 110 °C for several hours. The solvent was removed in vacuo, after which conc. sodium bicarbonate (100 mL) and EtOAc (20 mL) were added and stirred vigorously at 70 °C for 5 h. The product was extracted three times into EtOAc (50 mL). The organic layers were combined, washed with brine, dried with anhydrous sodium sulfate, and filtered. The solvent was removed in vacuo. The product was purified via silica gel column chromatography using PE/EtOAc (7:3). The title compound was isolated as an orange powder (197.0 mg, 0.48 mmol, 51%).

**MP** 156.8–174.6 °C. **^1^H NMR** (600 MHz, CDCl_3_) δ 10.54 (s, 1H), 7.82 (d, *J* = 4.3 Hz, 1H), 7.39–7.31 (m, 2H), 7.14 (s, 2H), 7.07 (d, *J* = 4.3 Hz, 1H), 6.98 (d, *J* = 2.2 Hz, 2H), 6.93 (d, *J* = 1.4 Hz, 1H), 6.93–6.91 (m, 2H), 4.90 (d, *J* = 5.4 Hz, 2H), 3.94 (s, 3H), 3.90 (s, 3H), 3.82 (s, 3H). **^13^C NMR** (151 MHz, CDCl_3_) δ 190.0, 159.3, 149.5, 149.3, 144.2, 138.7, 135.2, 131.0, 129.7 (2C), 128.9, 128.8, 127.5, 121.1, 114.2 (2C), 111.8, 111.6, 56.1, 56.0, 55.3, 48.2. **HR-MS** (ESI-Q-TOF): m/z[M+H]^+^ C_22_H_23_N_3_O_3_S Calculated: 410.1532. Measured: 410.1511.

#### 3.1.9. 7-(3,4-Dimethoxyphenyl)-*N*-(4-methoxybenzyl)isothiazolo[4,3-b]pyridin-3-amine (**28**)

To a vial with a screw cap equipped with a stir bar were added **27** (190.0 mg, 0.46 mmol, 1.0 equiv.), MeOH (5 mL), and DCM (25 mL). Under stirring at room temperature, 30% H_2_O_2_ in water (0.200 mL) was added dropwise. The vial was capped and stirred at 40 °C for 18h. Water (100 mL) was added, and the product was extracted three times with DCM (100 mL). The organic layers were combined and washed once with brine and then dried with anhydrous sodium sulfate and filtered. The solvent was removed in vacuo, and the residue was purified via silica gel column chromatography using PE/EtOAc (6:4–0:10 in steps of 10% every 50 mL). The title compound was isolated as a yellow powder that fluoresced green–yellow in solution under UV light (160.0 mg, 0.393 mmol, 85%).

**MP** 161.8–168.2 °C. **^1^H NMR** (600 MHz, CDCl_3_) δ 8.36 (d, *J* = 4.2 Hz, 1H), 7.73 (d, *J* = 2.0 Hz, 1H), 7.68 (dd, *J* = 8.4, 2.0 Hz, 1H), 7.40–7.34 (m, 2H), 7.32 (d, *J* = 4.2 Hz, 1H), 7.01 (d, *J* = 8.4 Hz, 1H), 6.95–6.90 (m, 2H), 6.52 (t, *J* = 5.4 Hz, 1H), 4.51 (d, *J* = 5.3 Hz, 2H), 3.96 (s, 3H), 3.94 (s, 3H), 3.82 (s, 3H). **^13^C NMR** (151 MHz, CDCl_3_) δ 171.3, 159.7, 152.6, 149.9, 148.9, 144.7, 139.8, 136.1, 129.5 (2C), 128.7, 127.7, 122.0, 120.7, 114.3 (2C), 112.4, 111.1, 56.0, 56.0, 55.3, 51.3. **HR-MS** (ESI-Q-TOF): m/z[M+H]^+^ C_22_H_21_N_3_O_3_S Calculated: 408.1376. Measured 408.1369.

#### 3.1.10. 7-(3,4-Dimethoxyphenyl)isothiazolo[4,3-b]pyridin-3-amine (**29**)

To a vial with a screw cap equipped with a stir bar was added **28** (140.0 mg, 0.34 mmol, 1.0 equiv.), followed by DCM (6.9 mL) and water (0.3 mL). To this was added DDQ (171.4 mg, 0.76 mmol, 2.2 equiv.) at room temperature, and then this was stirred for 6 h at room temperature. A conc. sodium bicarbonate solution was added, and the product was extracted three times into DCM (50 mL). The organic layers were combined and washed with a conc. sodium bicarbonate solution and a conc. brine solution and then dried with anhydrous sodium sulfate and filtered. The solvent was removed in vacuo. The title compound was purified via silica gel column chromatography using PE/EtOAc (3:7) and isolated as a brown powder that fluoresced green-yellow in solution under UV-light (43.0 mg, 0.15 mmol, 44%).

**MP** 201.3–205.1 °C. **^1^H NMR** (600 MHz, CDCl_3_) δ 8.45 (d, *J* = 4.2 Hz, 1H), 7.72 (d, *J* = 2.0 Hz, 1H), 7.69 (dd, *J* = 8.3, 2.1 Hz, 1H), 7.32 (d, *J* = 4.2 Hz, 1H), 7.02 (d, *J* = 8.3 Hz, 1H), 5.62 (s, 2H), 3.97 (s, 3H), 3.95 (s, 3H). **^13^C NMR** (151 MHz, CDCl_3_) δ 169.5, 152.9, 150.1, 148.9, 145.9, 140.3, 137.0, 128.4, 122.1, 120.5, 112.5, 111.2, 56.1, 56.0. **HR-MS** (ESI-Q-TOF): m/z[M+H]^+^ C_14_H_13_N_3_O_2_S Calculated: 288.0801. Measured: 288.0808.

#### 3.1.11. 4-(7-(3,4-Dimethoxyphenyl)isothiazolo[4,3-b]pyridin-3-yl)morpholine (**12**)

To an oven-dried vial with a screw cap equipped with a stir bar was added **29** (20.0 mg, 69.6 µmol, 1.4 equiv.), followed by anhydrous K_2_CO_3_ (19.2 mg, 139.2 µmol, 2.9 equiv.). The vial was purged with nitrogen gas. Dry nitrogen-purged DMF (15.0 mL) was then added. Bis(2-bromoethyl)ether (11.3 mg, 48.7 µmol, 1.0 equiv.) was added via a syringe. The vial was placed in a hot oil bath at 100 °C for 3 h. Water was added, and the product was extracted three times into EtOAc (50 mL). The organic layers were washed three times with water (15 mL) and once with brine (15 mL) and then dried with anhydrous sodium sulfate and filtered. The solvent was removed in vacuo, and the residue was purified via silica gel column chromatography using PE/EtOAc (4:6). The title compound was isolated as an orange powder that fluoresced green–yellow in solution under UV light (9.0 mg, 25.2 µmol, 52%).

**MP** 156.2–159.8 °C. **^1^H NMR** (600 MHz, CDCl_3_) δ 8.42 (d, *J* = 4.2 Hz, 1H), 7.65–7.59 (m, 2H), 7.26 (d, *J* = 4.0 Hz, 1H), 7.01 (d, *J* = 8.1 Hz, 1H), 3.99–3.93 (m, 14H). **^13^C NMR** (151 MHz, CDCl_3_) δ 173.8, 154.7, 149.9, 148.9, 144.8, 140.4, 136.6, 128.6, 122.2, 120.2, 112.5, 111.2, 66.3 (2C), 56.04, 56.01, 50.6 (2C). **HR-MS** (ESI-Q-TOF): m/z[M+H]^+^ C_18_H_19_N_3_O_3_S Calculated: 358.1220. Measured: 358.1219.

### 3.2. GAK Affinity Studies

The GAK affinity studies were performed by Thermo Fisher Scientific (SelectScreen services), as described before [27]. Briefly, compounds **8**, **10**, and **12** were evaluated in a LanthaScreen™ GAK binding assay in which 10 titrations of the test compound solubilized in DMSO were transferred to a 384-well plate. This was followed by the addition of the kinase buffer (50 mM HEPES pH 7.5, 0.01% BRIJ-35, 10 mM MgCl, and 1 mM EGTA), the 2X kinase antibody (Eu Anti GST) mixture, and the 4X Tracer 222 solution. After shaking for 30 s and incubation for one hour at room temperature, the plate was read on a fluorescence plate reader. When the bound tracer in the active site was displaced by the test compound, fluorescence was not observed. The collected data were compared to a 0% displacement control with pure DMSO and a 100% displacement control with staurosporine, a known inhibitor of GAK, and were plotted against the logarithmic concentration parameter. The IC_50_ values were then extracted and calculated.

### 3.3. X-ray Crystallography

For the X-ray crystallography, a suitable crystal of compound **14** was selected, and intensity data were collected at 293(2) K on an Agilent SuperNova diffractometer with an Eos CCD detector using MoKα radiation. Using Olex2 [41], the structure was solved with the SHELXT [42] structure solution program using intrinsic phasing and refined with the SHELXL [43] refinement package using full-matrix least-squares minimization on F^2^. Non-hydrogen atoms were refined anisotropically, and hydrogen atoms were refined in the riding mode with isotropic temperature factors fixed at 1.2 times U_eq_ of the parent atoms (1.5 for the methyl groups). The crystal data, data collection, and structure refinement details are summarized in Table S1.

### 3.4. Molecular Modeling

Dockings were performed with Autodock Vina 1.2.3 [44]. PDB entry 4Y8D [26] (human GAK kinase) was prepared for docking in the ATP-binding site. The resulting figures were made using UCSF Chimera [45].

## 4. Conclusions

In order to expand the SAR of isothiazolo[4,3-*b*]pyridines as GAK inhibitors, a 3,7-disubstituted isothiazolo[4,3-*b*]pyridine was needed. To access this unexplored chemotype, a novel synthetic procedure was developed, starting from 2,4-dichloro-3-nitropyridine as a building block. A regio-selective C-4 Suzuki arylation reaction and palladium-catalyzed aminocarbonylation were the key steps in this synthetic sequence. Unlike the 3,5- and 3,6-disubstituted isomers, the 3-*N*-(morpholinyl)-7-(3,4-dimethoxyphenyl)-isothiazolo[4,3-*b*]pyridine was completely inactive as a GAK inhibitor. Molecular modeling demonstrated that the substituent at position 7 prevented effective interactions between the isothiazole moiety and the kinase hinge region.

## Figures and Tables

**Table 1:** Palladium-catalyzed aminocarbonylation of compound **14** using CO and benzylamine.

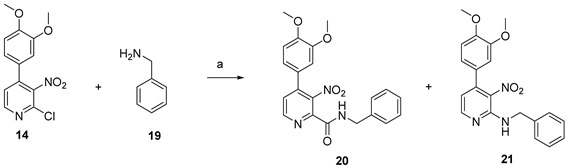				
Entry	Equivalents of CO	Catalyst Loading	Yield of 20	Yield of 21
1	1.3	1 mol%	23%	15%
2	2.6	1 mol%	42%	50%
3	2.6	2.5 mol%	78%	15%

Reaction conditions: chamber A: Na_2_CO_3_, Pd(OAc)_2_, XantPhos, toluene; and chamber B: HCOOH, MsCl, Et_3_N, toluene; 100 °C, 2.5 h.

**Table 2:** GAK affinity data of isothiazolo[4,3-*b*]pyridines **8**, **10**, and **12**.

Compound	3,5-disubstituted isothiazolo[4,3-*b*]pyridine **10**	3,6-disubstituted isothiazolo[4,3-*b*]pyridine **8**	3,7-disubstituted isothiazolo[4,3-*b*]pyridine **12**
Structure	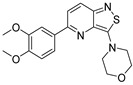	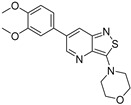	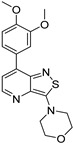
GAK IC_50_ (µM)	0.124 [28]	0.442	>10

## Data Availability

Crystallographic data for compound **14** have been deposited with the Cambridge Crystallographic Data Centre as supplementary publication number CCDC 2308765.

## Supplementary Materials

The following supporting information can be downloaded at https://www.mdpi.com/article/10.3390/molecules29050954/s1: Table S1: Crystal data and structure refinement for compound **14**. Figures S1–S21: Copies of NMR spectra of the intermediates and the final compound.
