# Supplementary material for: Synthesis of a 3,7-Disubstituted Isothiazolo[4,3-b]pyridine as a Potential Inhibitor of Cyclin G-Associated Kinase
Source: Molecules. 2024 Feb 22;29(5):954. doi: 10.3390/molecules29050954 (PMC10934046; doi:10.3390/molecules29050954)

Supporting Information

Synthesis of a 3,7-Disubstituted Isothiazolo[4,3-*b*]pyridine as a Potential Inhibitor of Cyclin G-Associated Kinase

Tom Grisez [1], Nitha Panikkassery Ravi [1], Mathy Froeyen [2], Dominique Schols [3], Luc Van Meervelt [4], Steven De Jonghe [3] and Wim Dehaen [1,*]

1 Department of Chemistry, Sustainable Chemistry for Metals and Molecules, KU Leuven, Celestijnenlaan 200F, B-3001 Leuven, Belgium; tom.grisez@kuleuven.be (T.G.); nitha.panikkasseryravi@kuleuven.be (N.P.R.)
2 Laboratory of Medicinal Chemistry, Rega Institute for Medical Research, Department of Pharmaceutical and Pharmacological Sciences, KU Leuven, Herestraat 49, P.O. Box 1041, B-3000 Leuven, Belgium; mathy.froeyen@kuleuven.be
3 Laboratory of Virology and Chemotherapy, Rega Institute for Medical Research, Department of Microbiology, Immunology and Transplantation, KU Leuven, Herestraat 49, P.O. Box 1043, B-3000 Leuven, Belgium; dominique.schols@kuleuven.be (D.S.); steven.dejonghe@kuleuven.be (S.D.J.)
4 Department of Chemistry, Biomolecular Architecture, KU Leuven, Celestijnenlaan 200F, B-3001 Leuven, Belgium; luc.vanmeervelt@kuleuven.be
* Correspondence: wim.dehaen@kuleuven.be

Contents

Table S1. Crystal data and structure refinement for compound **(14).** S2
Figure S1. ^{1}H NMR (400 MHz, $CDCl_3$) of compound **(14)**. S3
Figure S2. ^{13}C NMR (101 MHz, $CDCl_3$) of compound **(14)**. S3
Figure S3. ^{1}H NMR (400 MHz, DMSO-d_6) of compound **(18).** S4
Figure S4. ^{1}H NMR (400 MHz, $CDCl_3$) of compound **(20).** S5
Figure S5. ^{13}C NMR (101 MHz, $CDCl_3$) of compound **(20)**. S5
Figure S6. ^{1}H NMR (400 MHz, $CDCl_3$) of compound **(21).** S6
Figure S7. ^{13}C NMR (101 MHz, $CDCl_3$) of compound **(21).** S6
Figure S8. ^{1}H NMR (600 MHz, $CDCl_3$) of compound **(23).** S7
Figure S9. ^{13}C NMR (151 MHz, $CDCl_3$) of compound **(23)**. S7
Figure S10. ^{1}H NMR (400 MHz, $CDCl_3$) of compound **(24).** S8
Figure S11. ^{13}C NMR (101 MHz, $CDCl_3$) of compound **(24).** S8
Figure S12. ^{1}H NMR (400 MHz, $CDCl_3$) of compound **(26).** S9
Figure S13. ^{13}C NMR (101 MHz, $CDCl_3$) of compound **(26)**. S9
Figure S14. ^{1}H NMR (600 MHz, $CDCl_3$) of compound **(27).** S10
Figure S15. ^{13}C NMR (151 MHz, $CDCl_3$) of compound **(27)**. S10
Figure S16. ^{1}H NMR (600 MHz, $CDCl_3$) of compound **(28).** S11
Figure S17. ^{13}C NMR (151 MHz, $CDCl_3$) of compound **(28).** S11
Figure S18. ^{1}H NMR (600 MHz, $CDCl_3$) of compound **(29).** S12
Figure S19. ^{13}C NMR (151 MHz, $CDCl_3$) of compound **(29).** S12
Figure S20. ^{1}H NMR (600 MHz, $CDCl_3$) of compound **(12).** S13
Figure S21. ^{13}C NMR (151 MHz, $CDCl_3$) of compound **(12).** S13

Table S1. Crystal data and structure refinement for compound (14).

Empirical formula	$C_{13}H_{11}ClN_2O_4$
Formula weight	294.69
Temperature/K	293(2)
Crystal system	monoclinic
Space group	$P2_1/c$
a/Å	9.2334(5)
b/Å	7.8518(3)
c/Å	19.3020(9)
α/°	90
β/°	102.257(5)
γ/°	90
Volume/Å^3	1367.47(11)
Z	4
ρ_{calc} g/cm^3	1.431
μ/mm^{-1}	0.294
F(000)	608.0
Crystal size/mm^3	0.5 × 0.4 × 0.25
Radiation	Mo Kα (λ = 0.71073 Å)
2Θ range for data collection/°	5.546 to 52.742
Index ranges	$-11 \leq h \leq 11$, $-9 \leq k \leq 9$, $-24 \leq l \leq 24$
Reflections collected	14291
Independent reflections	2786 [R_{int} = 0.0309, R_{sigma} = 0.0214]
Data/restraints/parameters	2786/0/184
Goodness-of-fit on F^2	1.036
Final R indexes [I>=2σ (I)]	R_1 = 0.0421, wR_2 = 0.0996
Final R indexes [all data]	R_1 = 0.0547, wR_2 = 0.1081
Largest diff. peak/hole / e Å^{-3}	0.28/-0.33
CCDC deposition code	2308765

Figure S1. ^{1}H NMR (400 MHz, $CDCl_3$) of compound **(14)**.

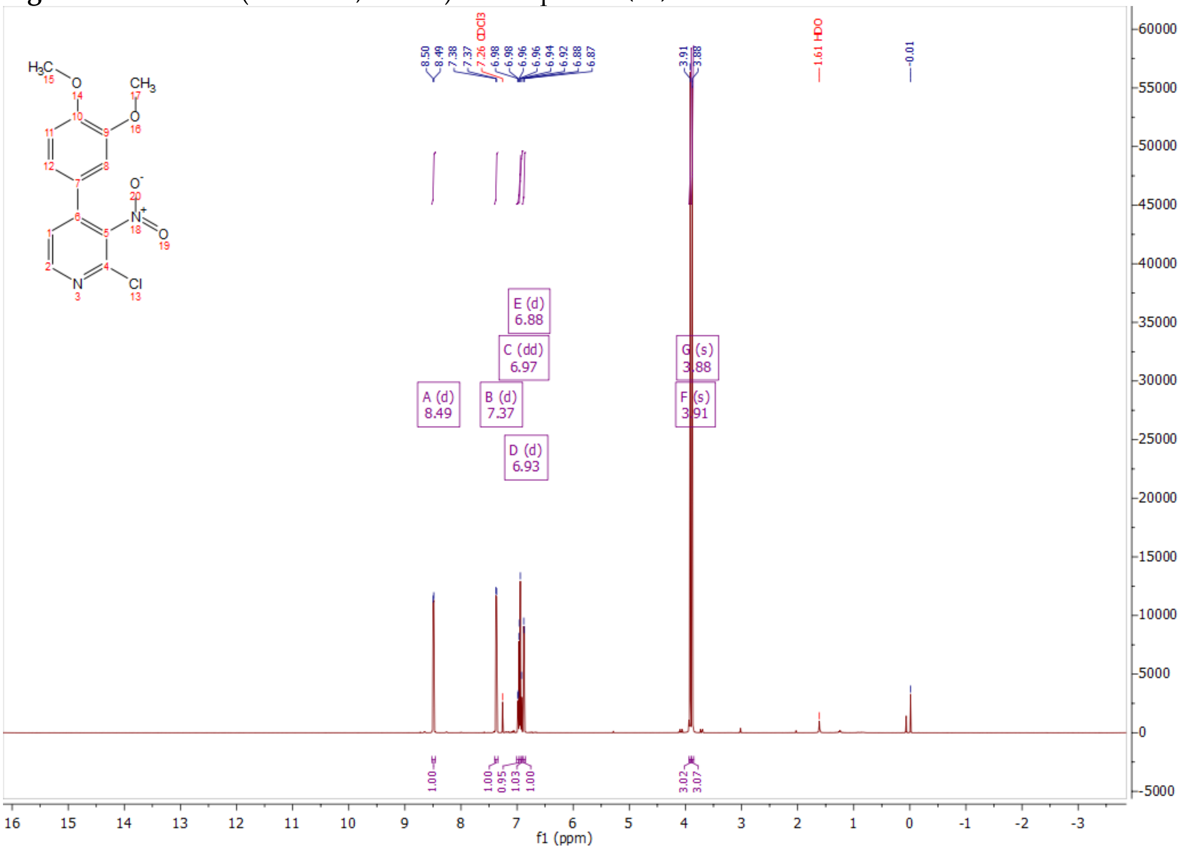

Figure S2. ^{13}C NMR (101 MHz, $CDCl_3$) of compound **(14)**.

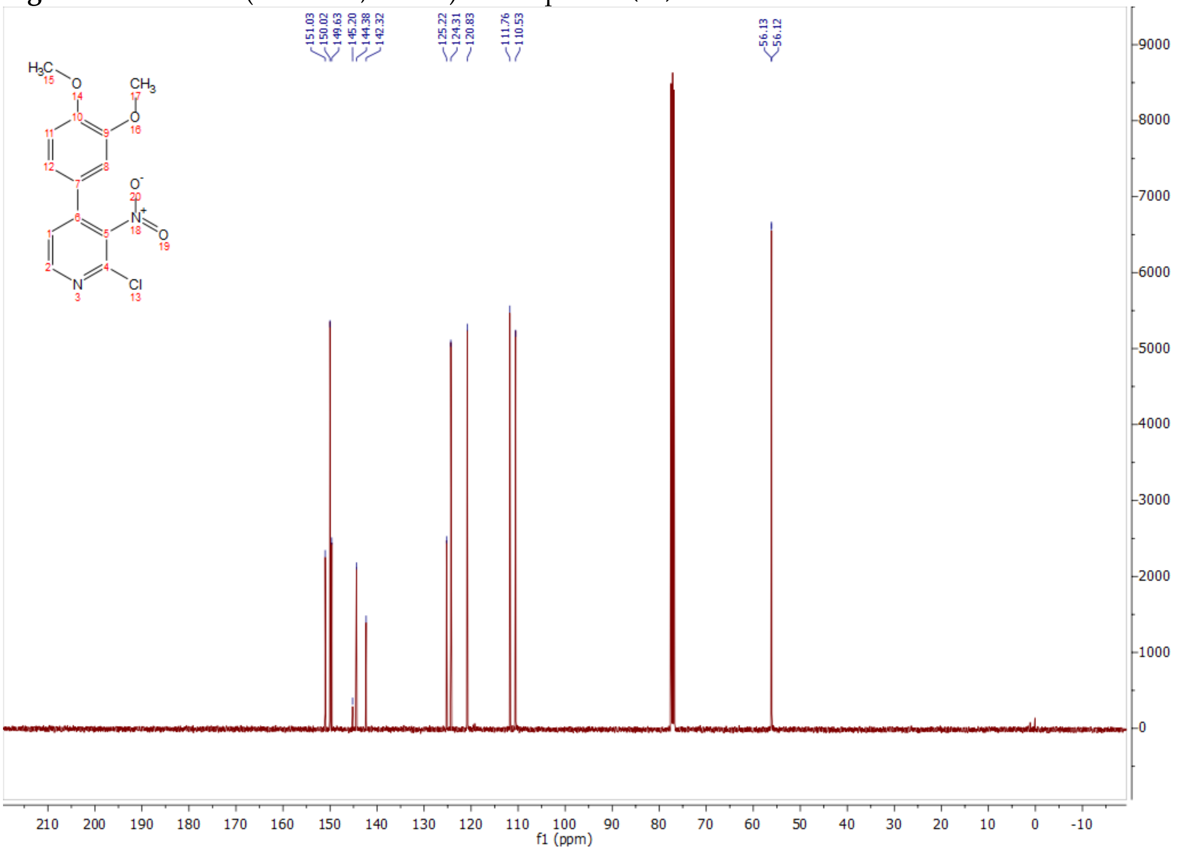

Figure S3. ^{1}H NMR (400 MHz, DMSO-d_6) of compound **(18).**

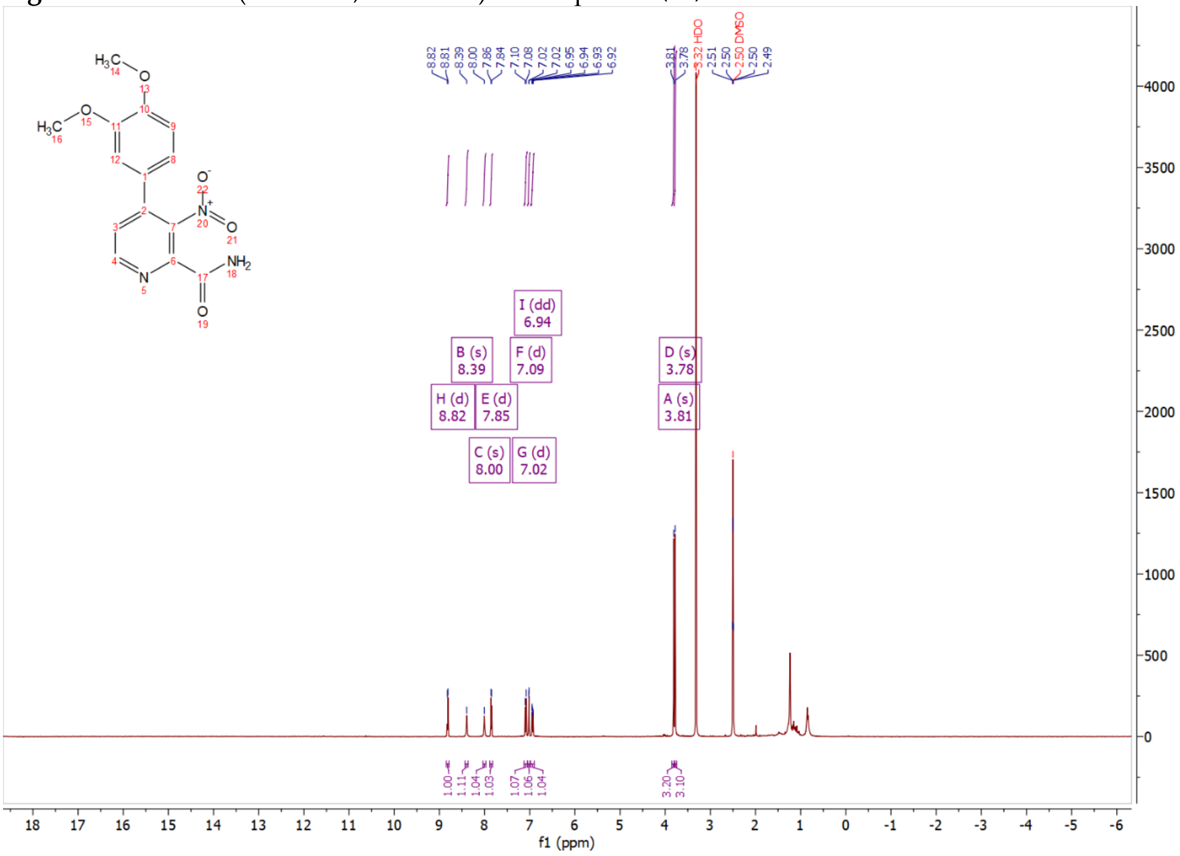

Figure S4. ^{1}H NMR (400 MHz, $CDCl_3$) of compound **(20).**

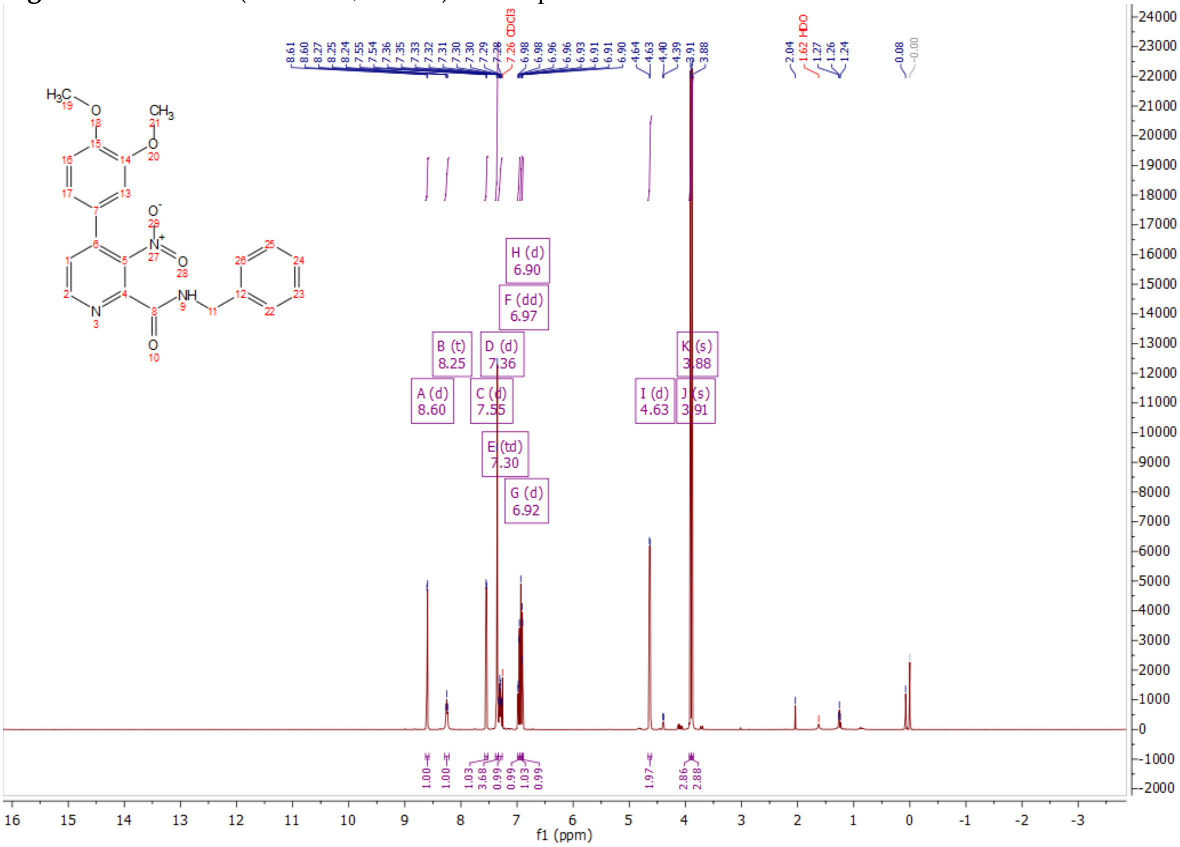

Figure S5. ^{13}C NMR (101 MHz, $CDCl_3$) of compound **(20)**.

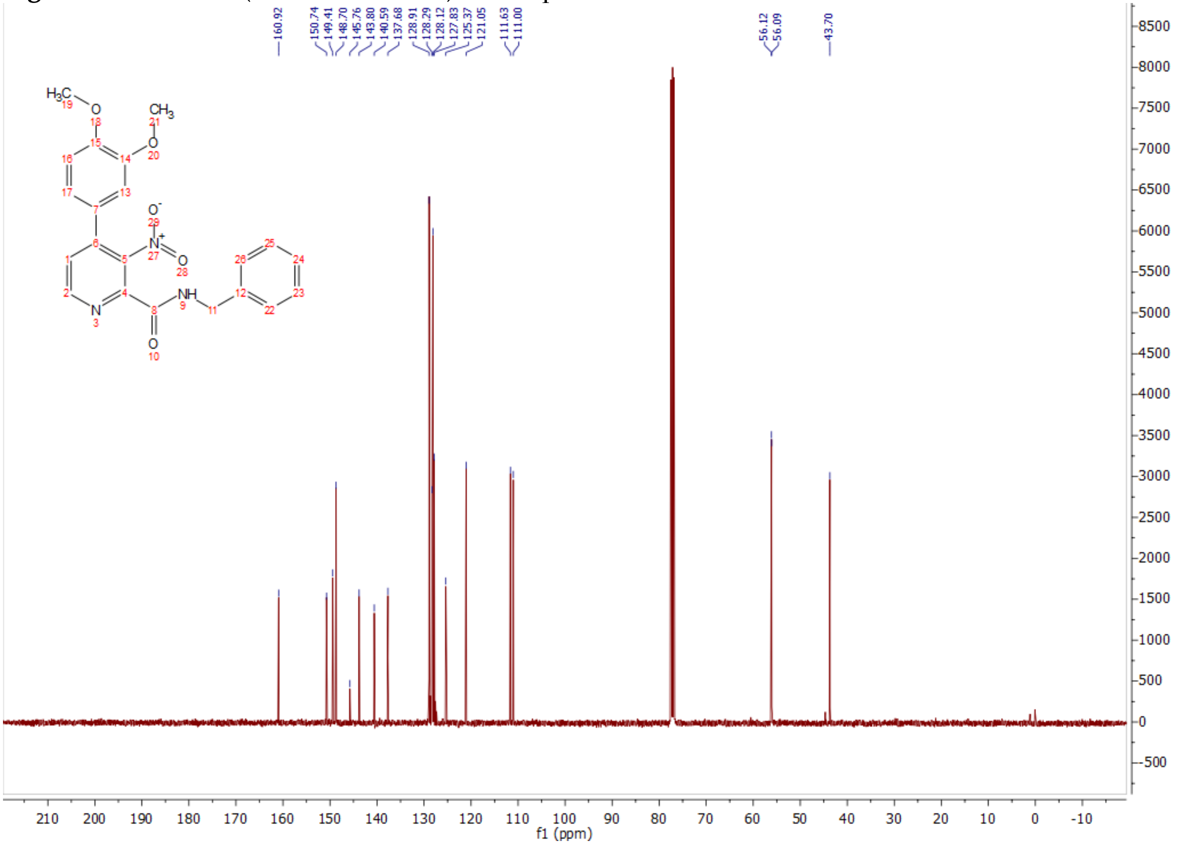

Figure S6. ^{1}H NMR (400 MHz, $CDCl_3$) of compound **(21).**

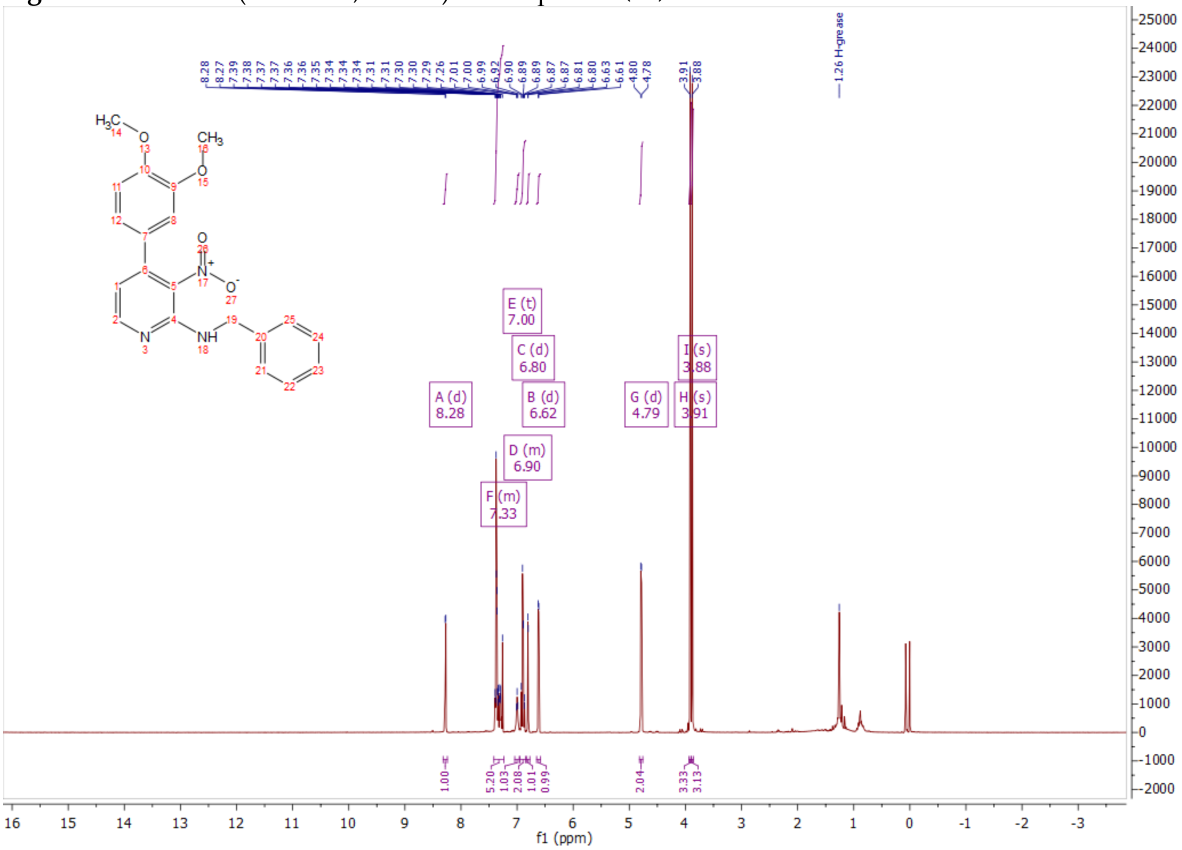

Figure S7. ^{13}C NMR (101 MHz, $CDCl_3$) of compound **(21).**

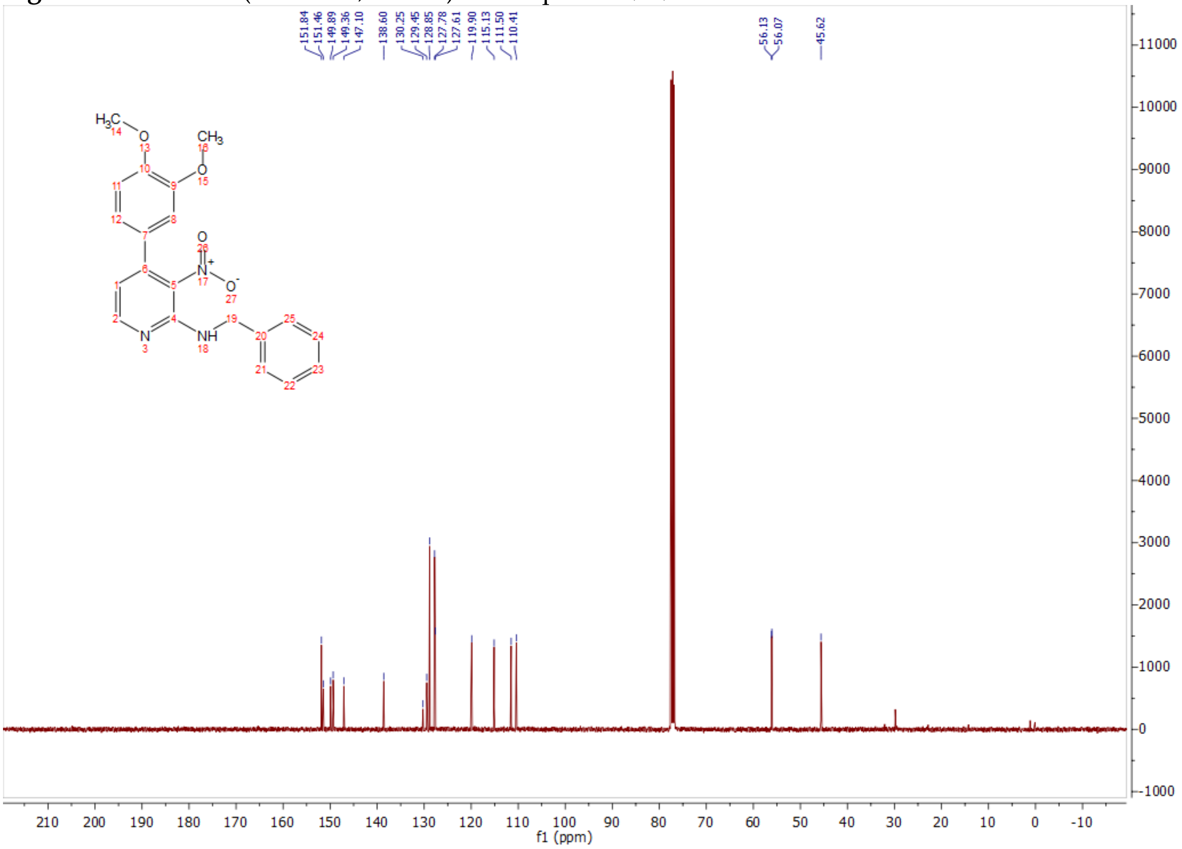

Figure S8. ^{1}H NMR (600 MHz, $CDCl_3$) of compound **(23).**

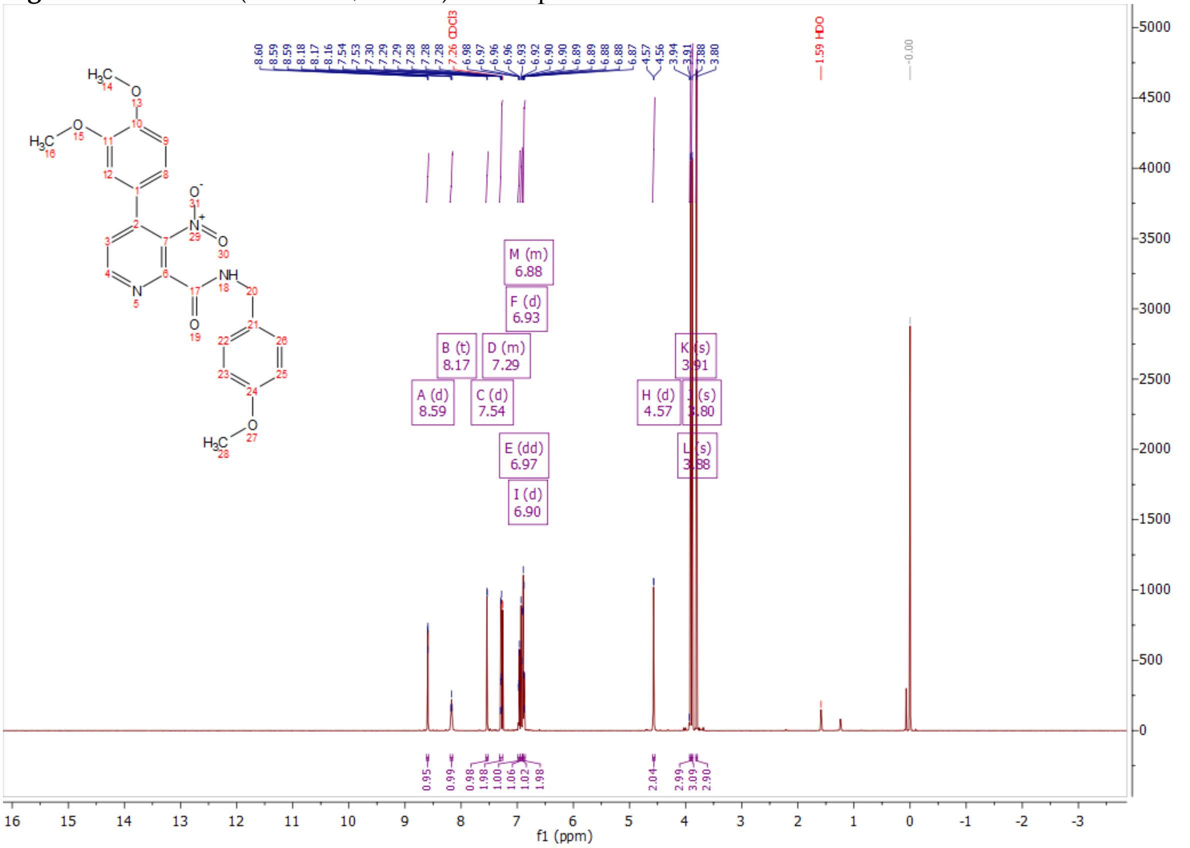

Figure S9. ^{13}C NMR (151 MHz, $CDCl_3$) of compound **(23)**.

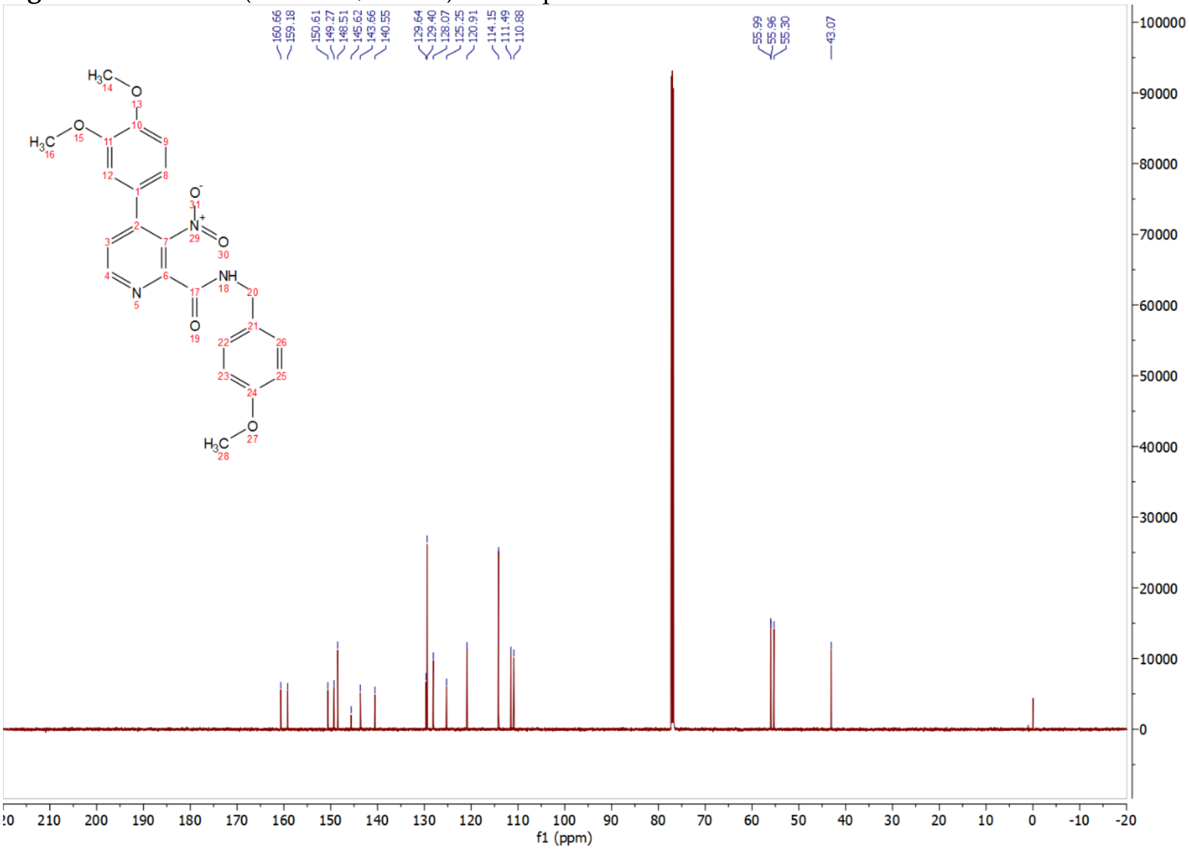

Figure S10. ^{1}H NMR (400 MHz, $CDCl_3$) of compound **(24).**

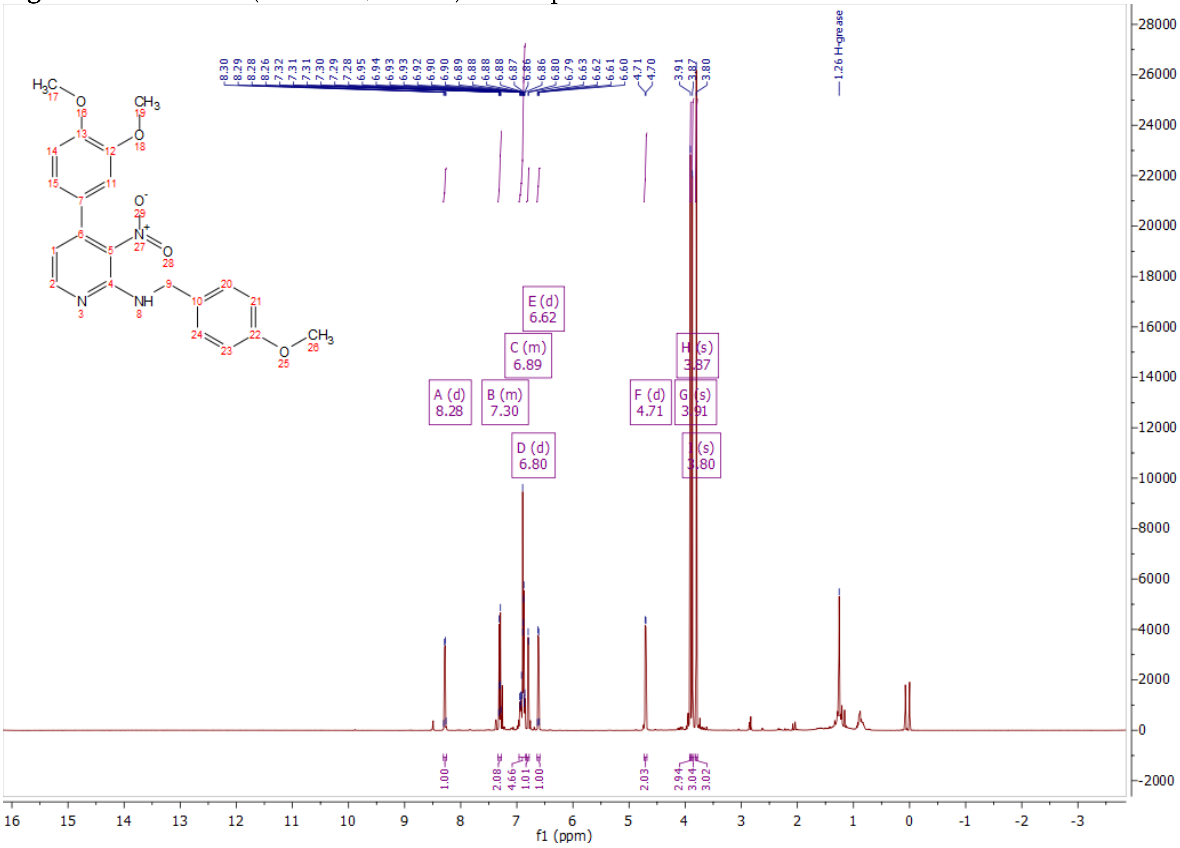

Figure S11. ^{13}C NMR (101 MHz, $CDCl_3$) of compound **(24).**

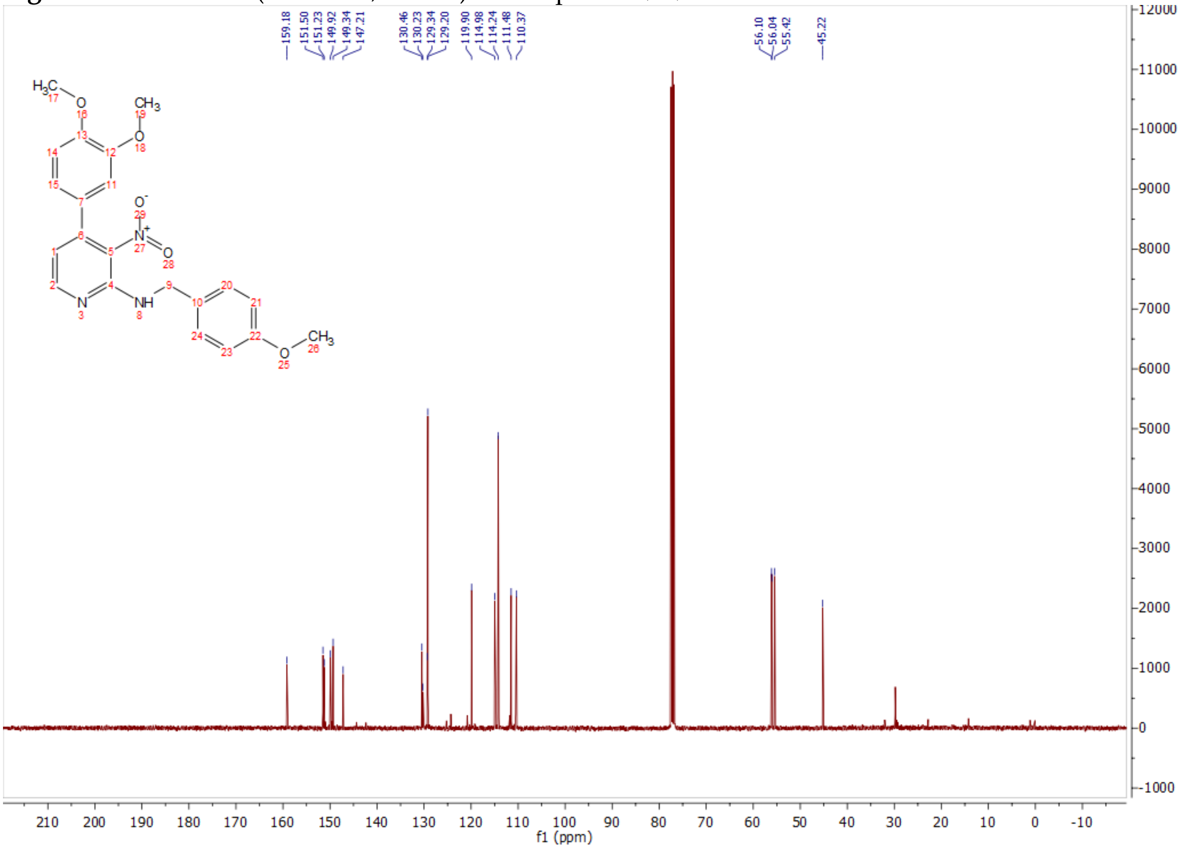

Figure S12. ^{1}H NMR (400 MHz, $CDCl_3$) of compound **(26).**

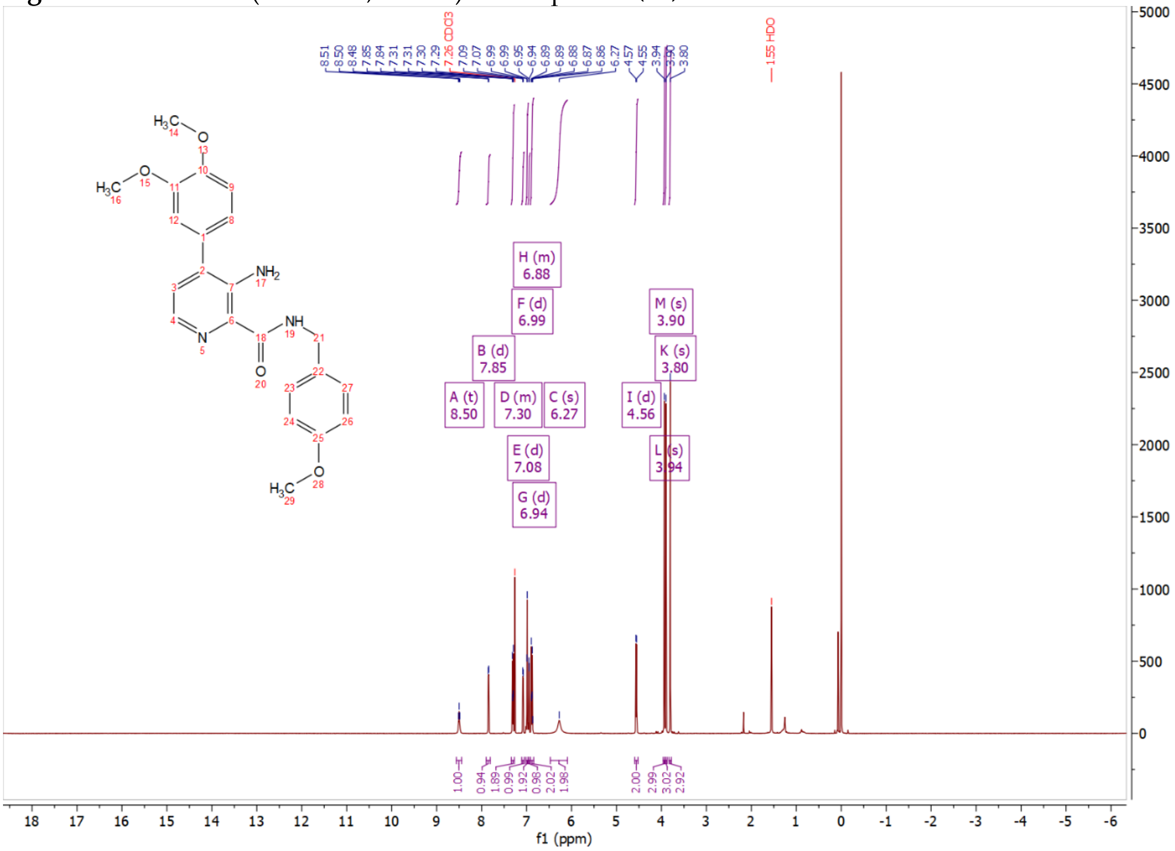

Figure S13. ^{13}C NMR (101 MHz, $CDCl_3$) of compound **(26)**.

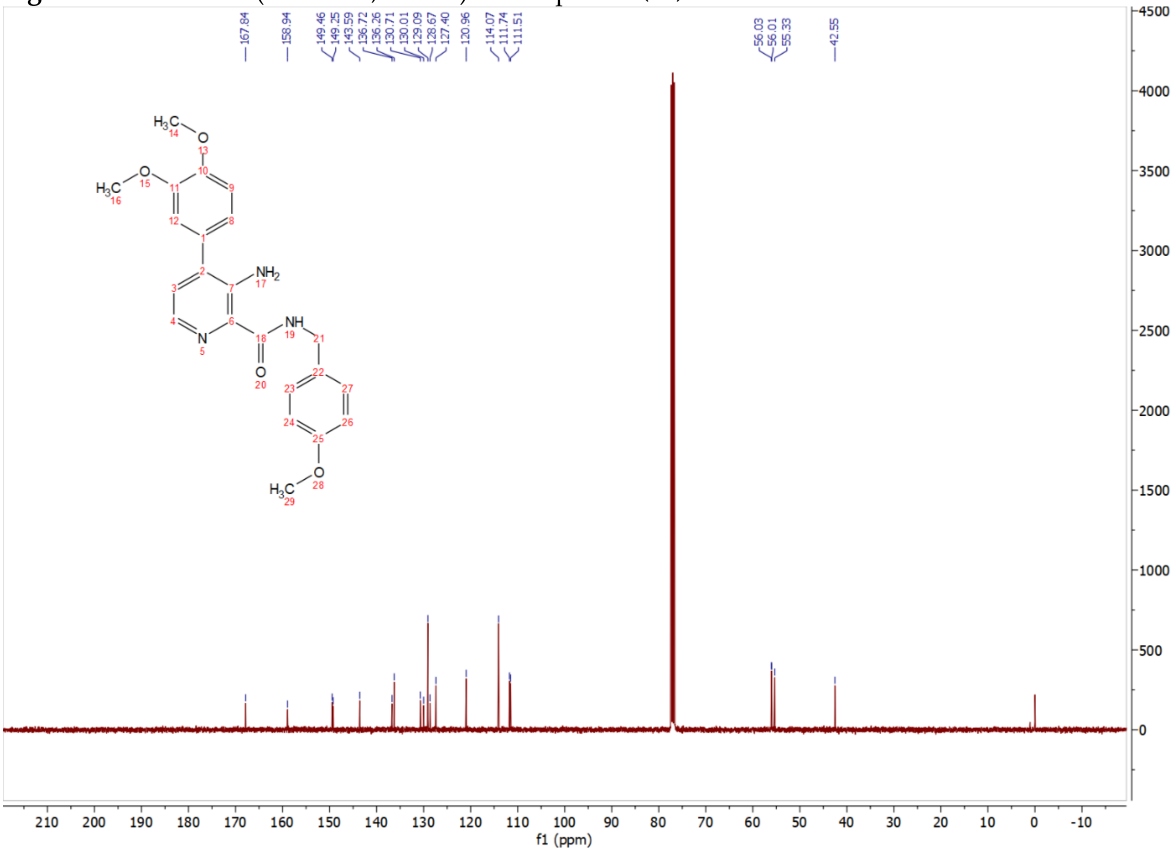

Figure S14. ^{1}H NMR (600 MHz, $CDCl_3$) of compound **(27).**

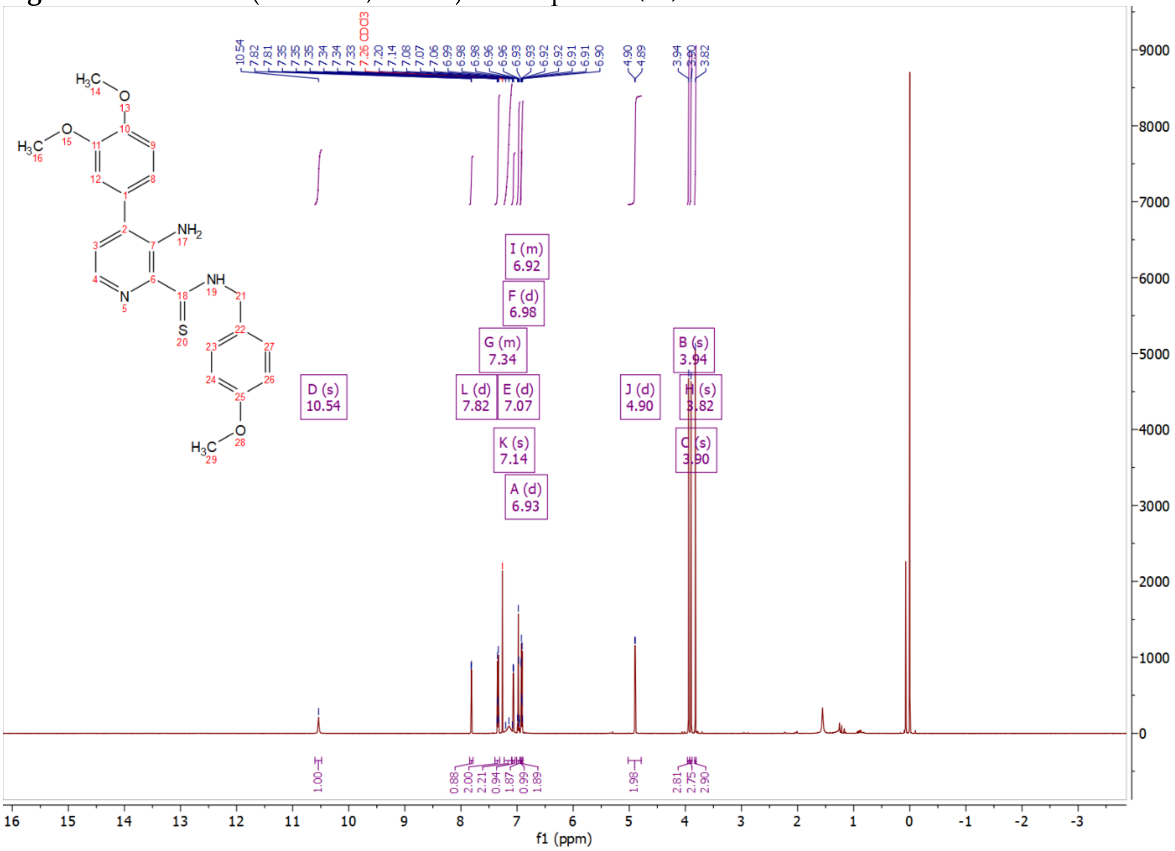

Figure S15. ^{13}C NMR (151 MHz, $CDCl_3$) of compound **(27)**.

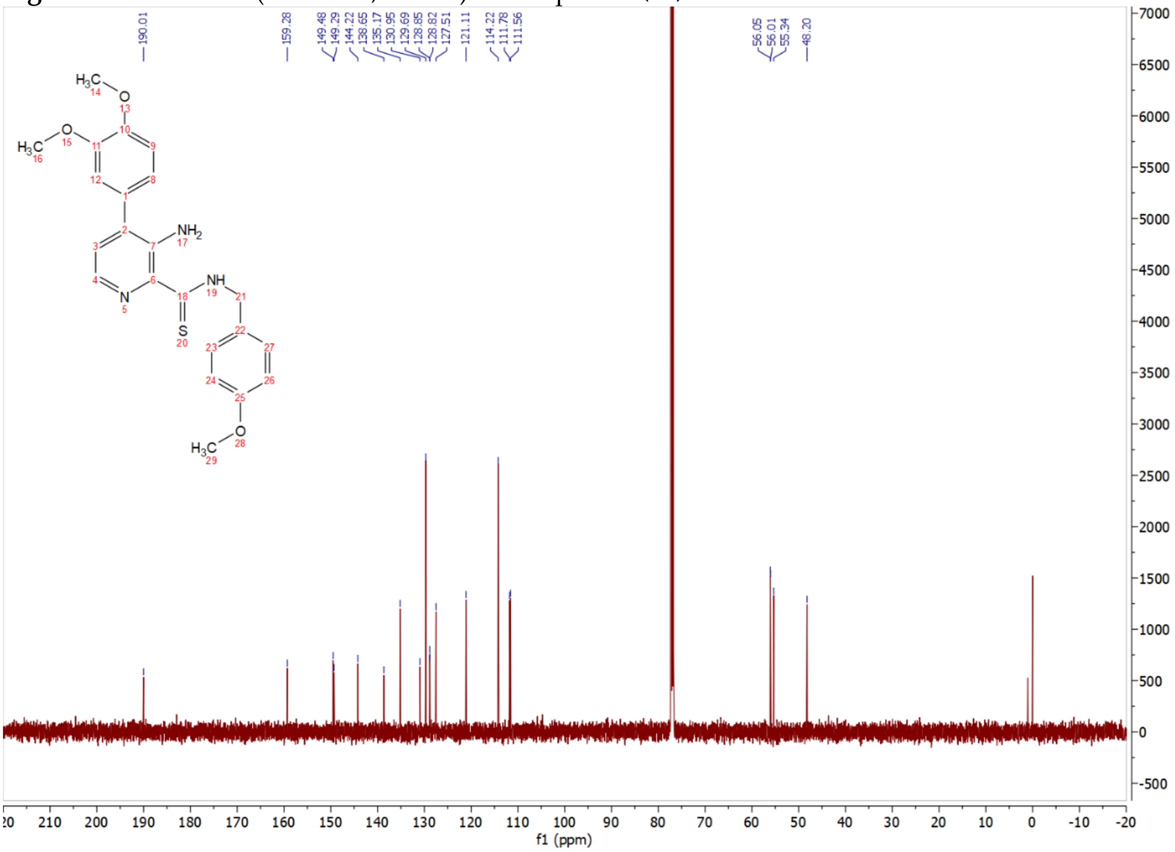

Figure S16. ^{1}H NMR (600 MHz, $CDCl_3$) of compound **(28).**

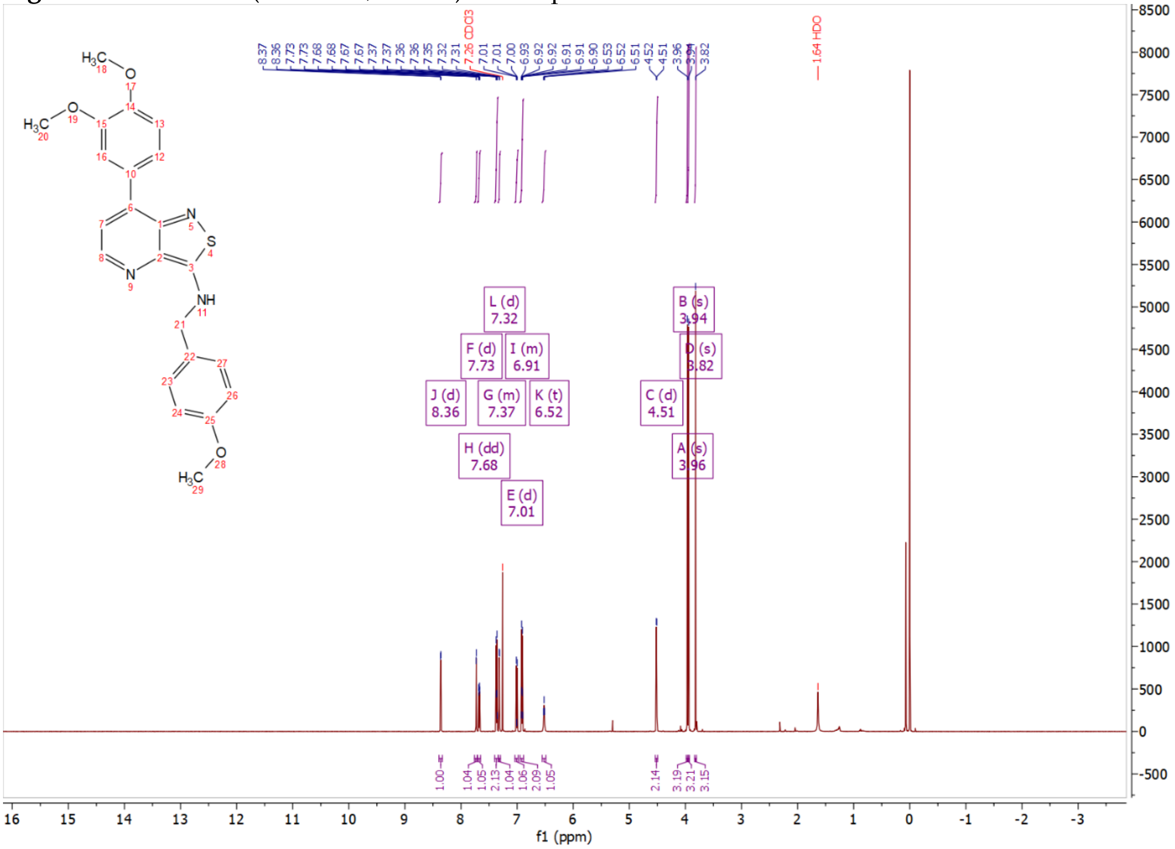

Figure S17. ^{13}C NMR (151 MHz, $CDCl_3$) of compound **(28).**

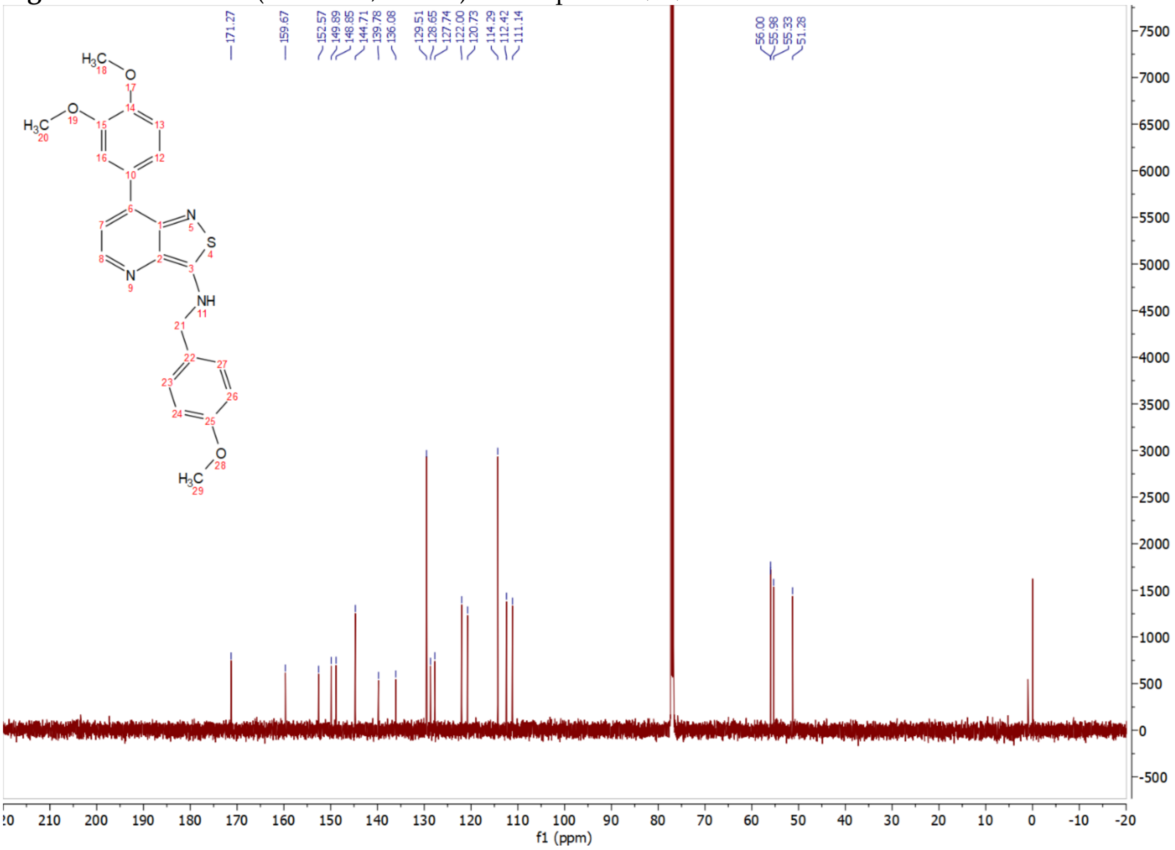

Figure S18. ^{1}H NMR (600 MHz, $CDCl_3$) of compound **(29).**

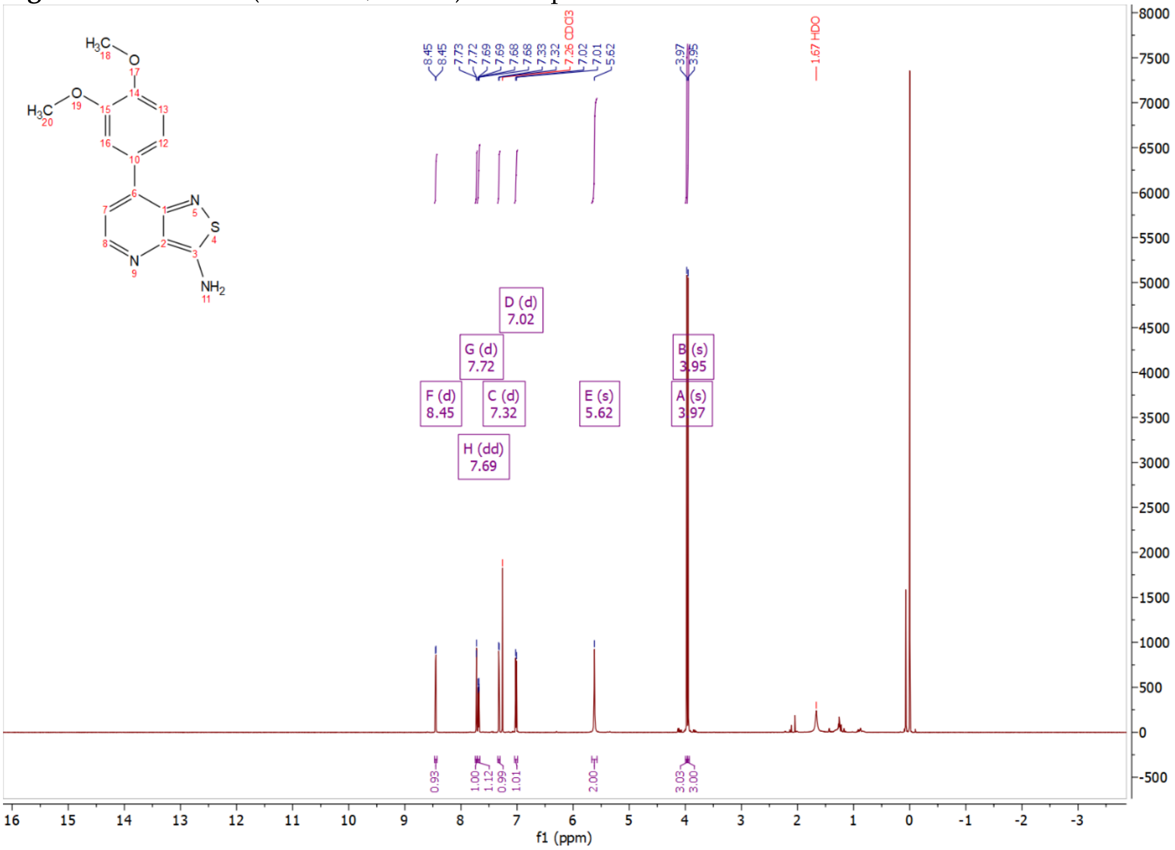

Figure S19. ^{13}C NMR (151 MHz, $CDCl_3$) of compound **(29).**

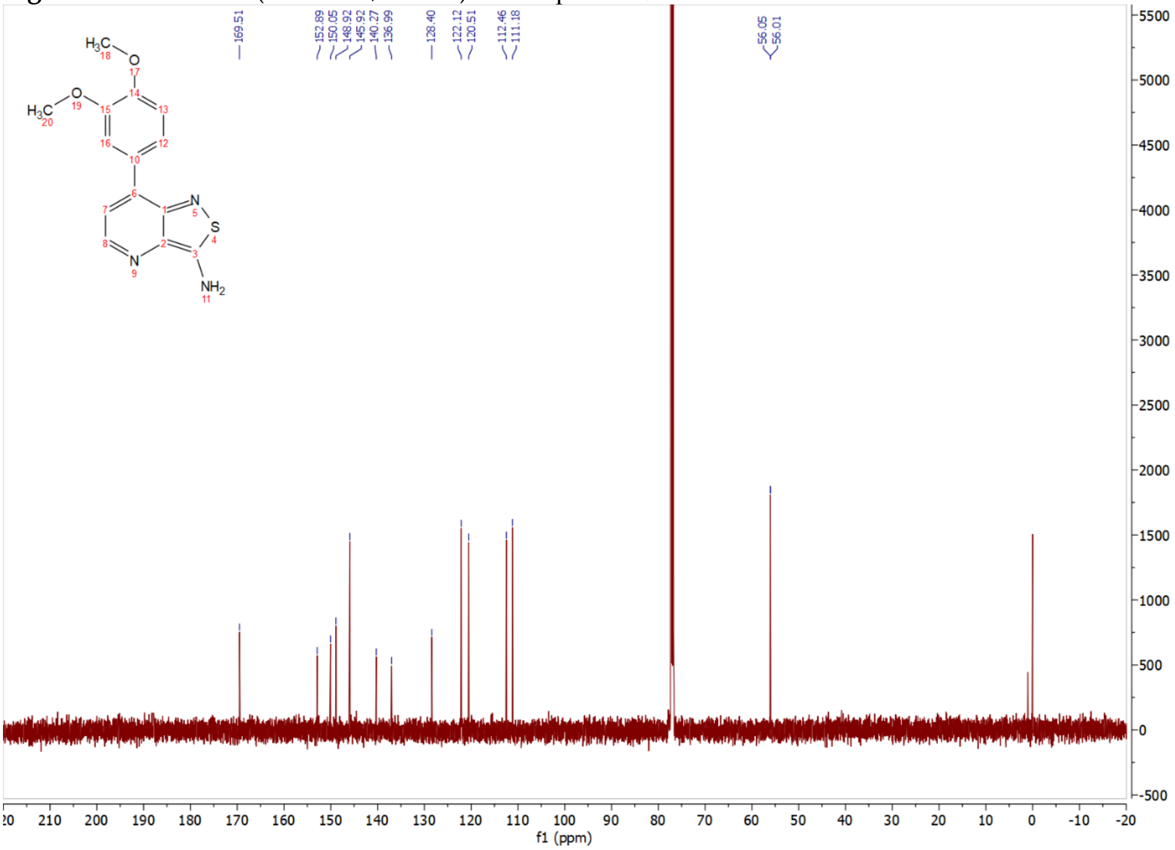

Figure S20. ^{1}H NMR (600 MHz, $CDCl_3$) of compound **(12).**

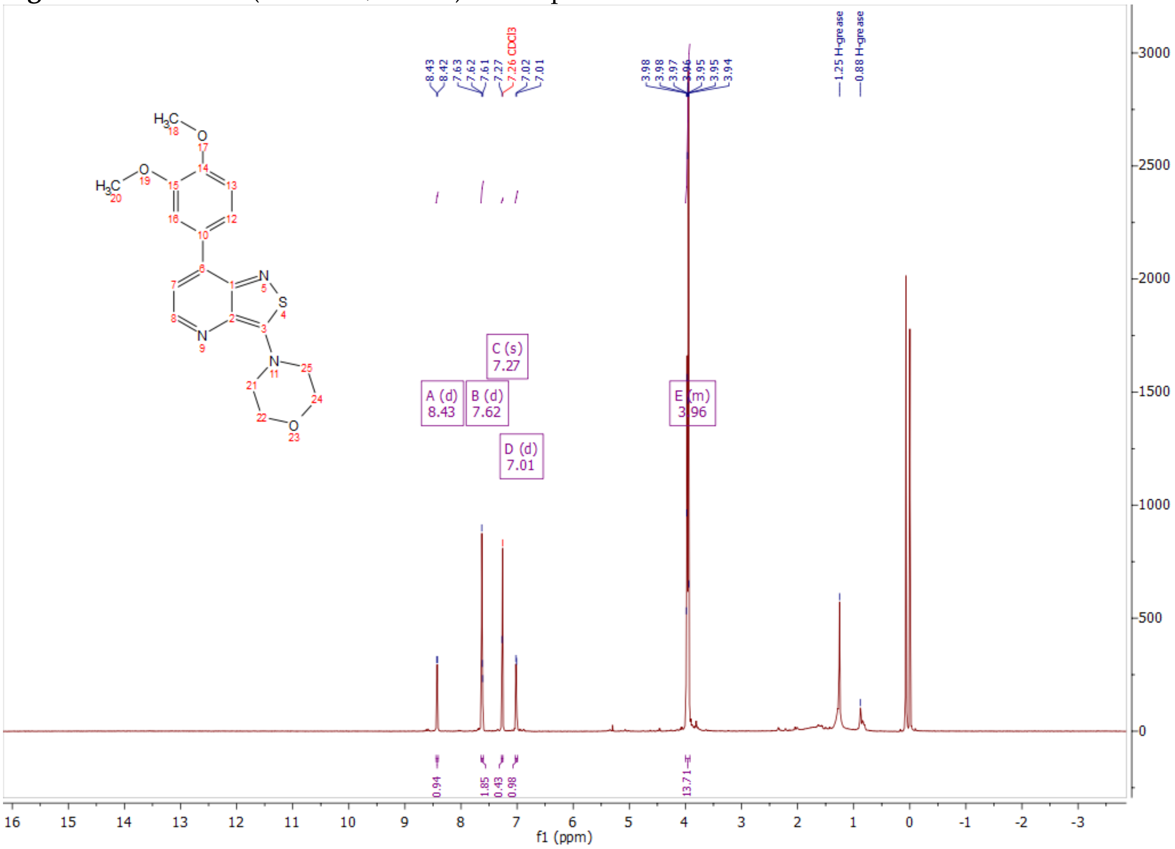

Figure S21. ^{13}C NMR (151 MHz, $CDCl_3$) of compound **(12).**

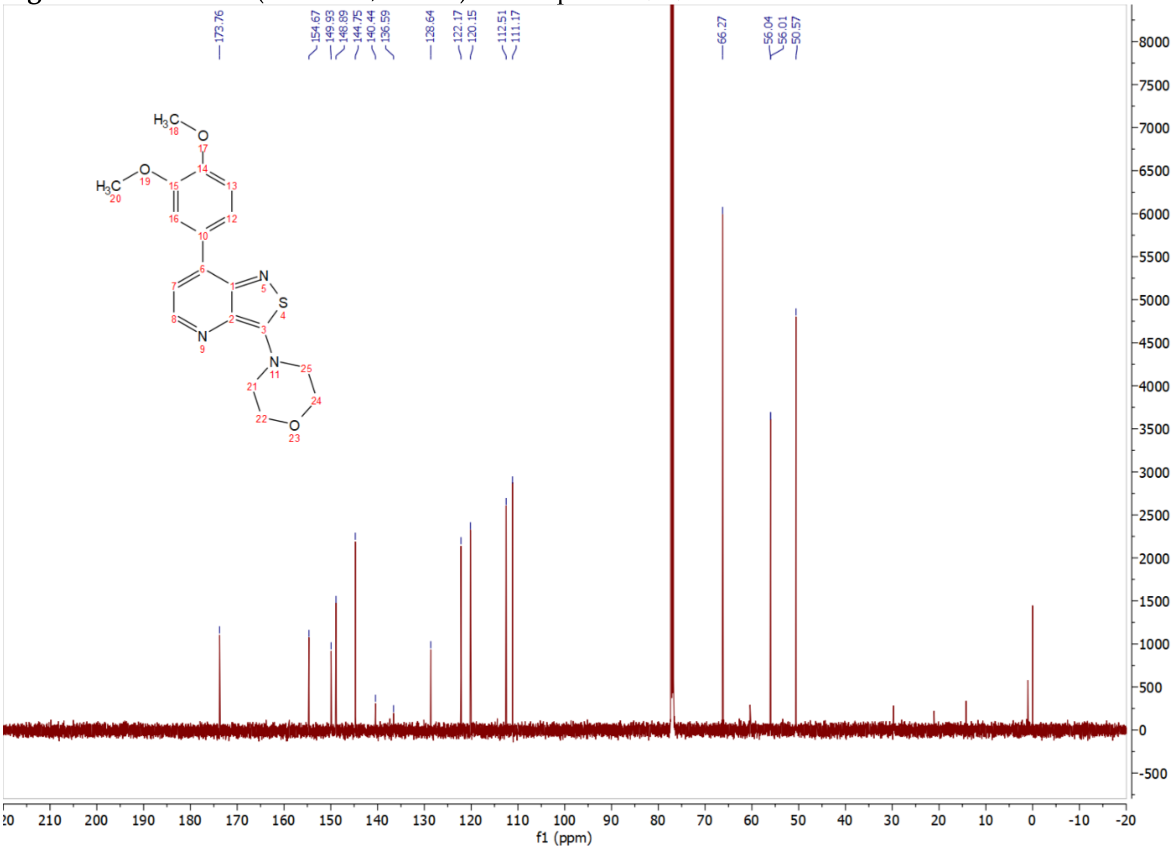

Supplement: Supplementary file 1 [file molecules-29-00954-s001.zip › molecules-2850294-supplementary.pdf]
